# Switchable Coacervate Formation via Amino Acid Functionalization of Poly(dehydroalanine)

**DOI:** 10.1021/acs.biomac.4c00048

**Published:** 2024-03-01

**Authors:** Casey A. Morrison, Ethan P. Chan, Thatcher Lee, Timothy J. Deming

**Affiliations:** † Department of Chemistry and Biochemistry, University of California, Los Angeles, Los Angeles, California 90095, United States; ‡ Department of Chemistry, Smith College, Northampton, Massachusetts 01063, United States; § Department of Bioengineering, University of California, Los Angeles, Los Angeles, California 90095, United States

## Abstract

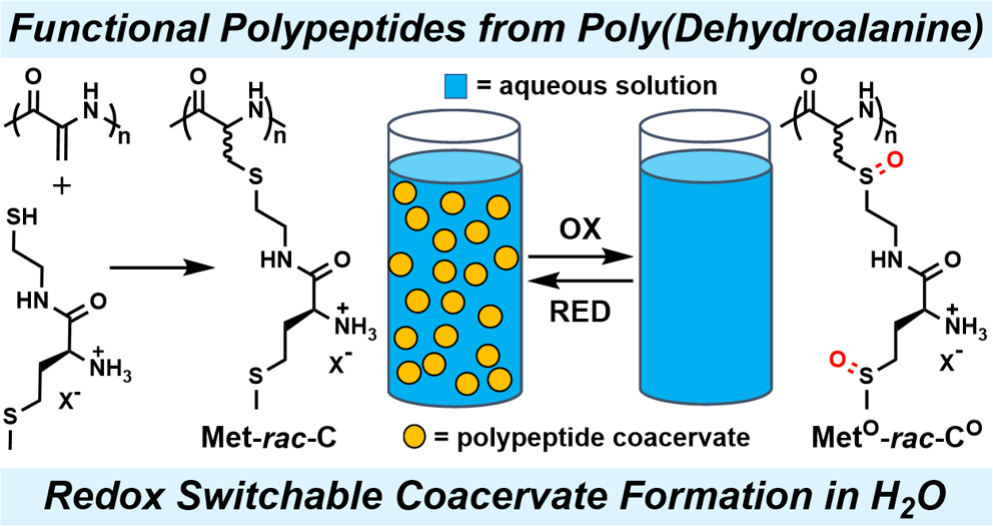

Our group recently developed a family of side-chain amino acid-functionalized poly(S-alkyl-l-homocysteines), **Xaa-C**^**H**^ (Xaa = generic amino acid), which possess the ability to form environmentally responsive coacervates in water. In an effort to further study how the molecular structure affects polypeptide coacervate formation, we prepared side-chain amino acid-functionalized poly(S-alkyl-*rac*-cysteines), **Xaa-*****rac*****-C**, via post-polymerization modification of poly(dehydroalanine), **A**^**DH**^. The use of the **A**^**DH**^ platform allowed straightforward synthesis of a diverse range of side-chain amino acid-functionalized polypeptides via direct reaction of unprotected l-amino acid 2-mercaptoethylamides with **A**^**DH**^. Despite their differences in the main-chain structure, we found that **Xaa-*****rac*****-C** can form coacervates with properties similar to those seen with **Xaa-C**^**H**^. These results suggest that the incorporation of side-chain amino acids onto polypeptides may be a way to generally favor coacervation. The incorporation of l-methionine in **Met-*****rac*****-C** allowed the preparation of coacervates with improved stability against high ionic strength media. Further, the presence of additional thioether groups in **Met-*****rac*****-C** resulted in an increased solubility change upon oxidation allowing facile reversible redox switching of coacervate formation in aqueous media.

## Introduction

**Scheme 1:**
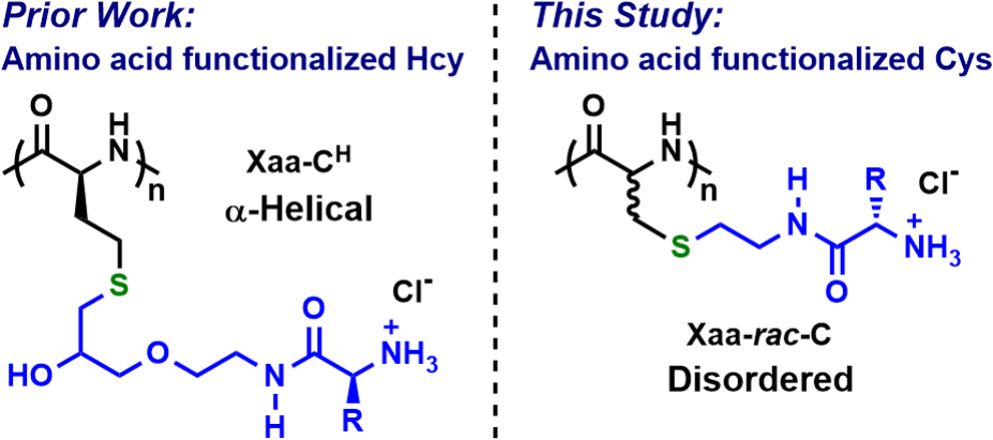
Comparison of α-Helical Polymers of Amino Acid-Functionalized Homocysteines (Hcy), **Xaa-C**^**H**^, to Disordered Polymers of Amino Acid-Functionalized Cysteines (Cys), **Xaa-*****rac*****-C** Xaa = generic amino acid.

Biomimetic coacervates are of interest as model systems to enable a better understanding of natural condensates as well as for the delivery of therapeutics and for the development of environmental stimuli-responsive biomaterials.^1−4^ For these uses, it is desirable that coacervate formation and properties can be adjusted at the molecular level, and that coacervation can also be readily switched on or off under physiologically relevant conditions.^5−9^ Many coacervate systems are either based on complex biopolymers (e.g., proteins or statistical copolymers) where structure–property relationships are not well understood leading to challenges in predictable tuning of properties^10−12^ or based on simple polymers (e.g., poly(l-lysine)) that lack the ability to respond to physiologically relevant stimuli.^13−15^ Our group recently developed a class of α-helical, side-chain amino acid-functionalized poly(S-alkyl-l-homocysteines), **Xaa-C**^**H**^, that possess the ability to form environmentally responsive coacervates in water (Scheme 1).^16^

Since they are compositionally uniform, **Xaa-C**^**H**^ homopolypeptides allow for a more detailed molecular understanding of structure–property relationships when polymer side-chain groups are altered, as compared to side-chain variation in statistical copolymers or protein sequences.^16^ We had previously found that hydrophobicity of side-chain amino acid groups could be varied to control coacervate versus precipitate formation and to adjust how temperature, pH, and counterion valency affect coacervate formation.^16^ Further, oxidation of thioether linkages in **Leu-C**^**H**^ was found to result in the formation of disordered conformations and increased polypeptide hydrophilicity, which acted as a mild, biomimetic switch to turn off coacervate formation via active control.^16^

Here, we sought to test if the incorporation of side-chain amino acid groups onto a different polypeptide backbone could also enable coacervate formation. These experiments were expected to aid understanding of how polypeptide chain conformation and flexibility influence coacervate formation and properties. Poly(dehydroalanine), **A**^**DH**^, was chosen as it can be prepared with controlled length, and it is readily post-polymerization functionalized under mild conditions in high yield by reaction with thiol nucleophiles.^17^ The products of such functionalization are poly(S-alkyl-*rac*-cysteines), which are expected to adopt disordered conformations in solution due to backbone heterochirality.^17^ Reactions of **A**^**DH**^ with unprotected L-amino acid 2-mercaptoethylamides were thus envisioned to give side-chain amino acid-functionalized poly(S-alkyl-*rac*-cysteines), **Xaa-*****rac*****-C**, which would possess side chains structurally similar to those in **Xaa-C**^**H**^ but with backbones that would be expected to adopt disordered instead of α-helical conformations (Scheme 1). In addition to the goal of creating new coacervate-forming polypeptides with tunable properties, we also sought to develop **Xaa-*****rac*****-C** polypeptide synthetic methodology since it has the potential for more straightforward preparation of a diverse range of polypeptides with highly functional side-chain groups as compared to our method for the synthesis of **Xaa-C**^**H**^.^16,17^

## Experimental Section

### Materials and Instrumentation

The following chemicals were used as received from vendors: l-cysteine hydrochloride monohydrate (Fisher), HOBT hydrate (Oakwood), *tert*-butyl bromoacetate (Fisher), triphosgene (Oakwood), iodomethane (Oakwood), HATU (Oakwood), N-BOC-l-amino acids (Oakwood), diisopropylethylamine (DIPEA) (Fisher), dichloromethane (DCM) (Fisher), ethyl acetate (Fisher), trifluoroacetic acid (TFA) (Fisher), 2,2′-bipyridine (bpy) (Sigma), and Ni(COD)_2_ (Strem). Tetrahydrofuran (THF) and hexanes were each degassed with dinitrogen and passed through an alumina column before use. All other reagents and solvents were used as received. In-house deionized water (DI H_2_O) was used for all aqueous chemistry and dialysis unless otherwise specified. bpyNiCOD, poly(l-lysine·HCl)_60_ (**K**_**60**_), (poly(*S*-(3-(2-l-leucine amido)ethoxy)-2-hydroxypropyl)-l-homocysteine hydrochloride)_60_ (**Leu-C^H^**_**60**_), and α-methoxy-ω-isocyanoethyl-poly(ethylene glycol), mPEG-NCO (*M*_n_ = 1 kDa) were prepared according to literature procedures.^16,18−20^ NCA purifications and polymerizations were performed in an N_2_-filled glovebox. Reactions at elevated temperatures were controlled using a Corning PC 420D thermostat-controlled hot plate equipped with a thermocouple probe. Room-temperature reactions were performed at *ca*. 20 °C ambient temperature. Small molecule chemistry was performed in heat-dried glassware under ambient atmosphere, unless otherwise specified. Unless otherwise specified, all post-polymerization modification chemistry was performed in glass vials under an ambient atmosphere. Thin-layer chromatography was performed with EMD gel 60 F254 plates (0.25 mm thickness), and spots were visualized using KMnO_4_ stain. Silicycle Siliaflash G60 silica (60–200 μm) was used for all column chromatography. Silica used for chromatographic purification of NCA monomers was dried under vacuum at 250 °C for 48 h and then stored in a dinitrogen-filled glovebox. Compositions of mobile phases used for chromatography are given in volume percentages. Dialysis was performed with regenerated cellulose tubing obtained from Spectrum laboratories with 1000 Da molecular weight cutoff (MWCO). NMR spectra of solution samples were recorded on Bruker AV400 and Bruker AV300 instruments with chemical shifts reported relative to deuterated solvent resonances. For the **C**^**BCM**^ precursor, gel permeation chromatography was performed at 40 °C with 1,1,1,3,3,3-hexafluoroisopropanol (HFIP) containing 0.5% (w/w) potassium trifluoroacetate (KTFA) eluent at a 1.0 mL/min flow rate using an LC-20AD Prominence HPLC Pump (Shimadzu) equipped with 100 and 1000 Å PSS-PFG 7 μm columns (PSS Agilent). Samples were prepared at 20 mg/mL and were detected using an RID-20A Refractive Index detector (Shimadzu). A calibration curve was prepared using monodisperse polyethylene glycol standards ranging from 1 to 40 kDa that were purchased from Scientific Polymer Products, Inc. For **Xaa-*****rac*****-C** samples, gel permeation chromatography was performed at 25 °C with trifluoroethanol (TFE) containing 20 mM sodium trifluoroacetate (NaTFA) eluent at a 0.5 mL/min flow rate using a JASCO PU-4180 LC pump system equipped with two 300 mm PFG analytical linear S 5 μm columns (PSS Agilent). Samples were prepared at 0.5 or 1.0 mg/mL and were detected using a JASCO UV-4075 detector. A calibration curve was prepared using monodisperse poly(methyl methacrylate) (PMMA) standards ranging from 1 to 60 kDa that were purchased from PSS Agilent. Samples for circular dichroism (CD) spectroscopy were prepared using DI H_2_O. CD spectra were collected using solutions of polypeptide (0.5 mg/mL) on a Chirascan VX spectrophotometer using a 0.1 cm path length quartz cuvette. Cloud point temperature measurements were recorded at a wavelength of 500 nm on an HP 8453 spectrophotometer equipped with an Agilent 8909A temperature control. ζ-potential data were collected using a Malvern Zetasizer NanoZS. Brightfield images were taken using a Zeiss Axio Observer Z1 optical microscope. FTIR spectroscopy was performed on a PerkinElmer Spectrum RX spectrometer.

#### General Procedure for Synthesis of L-Amino Acid 2-Mercaptoethylamides^21,22^

N-Boc protected amino acids (2.0 g, 1.0 equiv) were dissolved in DCM (30 mL) followed by the sequential addition of solid HOBT (1.1 equiv) and HATU (1.0 equiv). The resulting solutions were stirred at room temperature for 20 min and then cooled to 0 °C. To the reaction mixtures, a suspension of cystamine dihydrochloride (0.50 equiv) in DCM (50 mL) followed by DIPEA (3.0 equiv) was added dropwise. The reactions were allowed to warm to room temperature and were stirred overnight. The resulting solutions were washed with saturated NaHCO_3_, 10% citric acid (aq), saturated NaHCO_3_, and then brine. The solutions were then dried with anhydrous sodium sulfate and concentrated under vacuum. The crude products were purified via flash column chromatography with hexanes/ethyl acetate (1:1) for Ala, Val, Leu, and Met or with DCM/MeOH (95:5) for Pro. Sample fractions were combined and concentrated under reduced pressure to give white solids. These samples were then dissolved into MeOH (50 mL) and placed in an ice bath and cooled to 0 °C. Under constant stirring, NaBH_4_ (4.0 equiv) was added to each sample. The reactions were allowed to continue for 30 min in the ice bath and then for 30 additional min at room temperature. The reactions were then concentrated under reduced pressure and diluted with ethyl acetate. The solutions were adjusted to pH 3 by the addition of 1 M HCl and left to stir for 15 min. The organic phases were washed 2× with brine, dried with sodium sulfate, and concentrated under reduced pressure to yield white solids. Finally, the Boc groups were removed by dissolving the solids into ethyl acetate followed by the addition of 12 M HCl until the concentration of HCl in ethyl acetate was 3 M. The products were left to stir at room temperature for 2 h and then concentrated under reduced pressure. The products were used without further purification.

#### General Procedure for Modification of **A^DH^**_**65**_ with l-Amino Acid 2-Mercaptoethylamides

A sample of**A^DH^_65_** was dissolved in DMSO at 20 mg/mL. The desired l-amino acid 2-mercaptoethylamide (5 equiv per dehydroalanine residue) was dissolved in minimal DI H_2_O (∼300 mg/mL), and the pH was adjusted to pH 7.0 with 5 M NaOH. The l-amino acid 2-mercaptoethylamide solution was then added to the **A^DH^_65_** solution followed by the addition of 2 M NaOH (1.5 equiv per dehydroalanine residue). DMSO was then added to give a final **A^DH^_65_** concentration of 10 mg/mL. The reaction mixture was allowed to stir at room temperature for 18 h. The resulting solution was transferred to a 1000 Da MWCO dialysis bag and dialyzed against pH 4 DI H_2_O for 1 day with 2 dialyzate changes and then pH 7 DI H_2_O for 1 day with 2 dialyzate changes. The solution was lyophilized to give the product as a white fluffy solid.

#### General Procedure for Oxidation of Thioether Groups in **Xaa-*****rac*****-C**_**65**_ Polypeptides

This procedure was adapted from a literature method.^23^ Into a scintillation vial containing **Xaa-*****rac*****-C**_**65**_ (25 mg) was added 70 wt % aqueous *tert*-butyl hydroperoxide (TBHP) (16 equiv per thioether group), a catalytic amount of camphorsulfonic acid (CSA) solution in DI water (0.2 equiv per thioether group, 20 mg/mL), and additional DI water to bring the final concentration of **Xaa-*****rac*****-C**_**65**_ to 20 mg/mL. The reaction mixture was stirred vigorously for 24 h at room temperature, and the solution was then transferred to a 2000 Da MWCO dialysis bag and dialyzed against DI H_2_O (3.5 L) containing sodium thiosulfate (1.2 g, 2.2 mM) to neutralize residual peroxide (48 h, 4 dialyzate changes), followed by 3 mM HCl (24 h, 3 dialyzate changes) and H_2_O (8 h, 3 dialyzate changes). The retentate was lyophilized to provide the sulfoxide product, **Xaa-*****rac*****-C^O^**_**65**_.

#### *In Situ* Oxidation and Reduction of **Met-*****rac*****-C**_**65**_(16)

A sample of **Met-*****rac*****-C**_**65**_ was dissolved in DI H_2_O (10 mg/mL) and was mixed with an equal volume of aqueous 26 mM TPP and 300 mM NaCl at pH 7.0. The resulting turbid coacervate suspension was then treated with 0.5 M sodium periodate solution (1.05 equiv per thioether group over 2 additions) while stirring at 0 °C. Complete dissolution of the coacervate was observed after 5 min. After 2 h, the solution was dialyzed against aqueous 150 mM NaCl and 13 mM TPP (24 h, 1 dialyzate change) to remove residual sodium periodate. The sample (ca. 5 mg/mL) was then transferred to a glass vial, and thioglycolic acid (150 equiv per sulfoxide group) and 100 μL of 0.1 M NaOH were added. After 24 h, additional thioglycolic acid (150 equiv per sulfoxide group) and 100 μL of 0.1 M NaOH were added. The reaction mixture (pH ca. 5–6) was allowed to stir at room temperature. After 4 days, the pH was increased to 7.0 by the addition of 12 M NaOH whereupon coacervate formation occurred. Spectral data for the *in situ* oxidized and reduced products matched those for **Met**^**O**^**-*****rac*****-C^O^**_**65**_ and **Met-*****rac*****-C**_**65**_, respectively.

#### General Procedure for Preparation of Polypeptide Coacervates^16^

Stock solutions of **Xaa-*****rac*****-C**_**65**_, **Met**^**O**^**-*****rac*****-**C^O^**_**65**_**, and **Leu-*****rac*****-**C^O^**_**65**_** (all with chloride counterions) were prepared at 6.0 mg/mL in DI H_2_O. Separate stock solutions of **Met**^**O**^**-*****rac*****-**C^O^**_**65**_** and **Leu-*****rac*****-**C^O^**_**65**_** were prepared at 10 mg/mL in DI H_2_O containing NaCl (300 mM) for mixing with polyadenylic acid (polyA). Stock solutions (24 mM) of sodium salts of different counterions (phosphate, pyrophosphate, or tripolyphosphate) were prepared in DI H_2_O containing NaCl (300 mM). A stock solution of 2× PBS buffer (300 mM) was also prepared, as well as a solution of 10 mg/mL polyA in DI H_2_O. A single stock solution (sodium salts, 2× PBS, or polyA) was then mixed with a polypeptide stock solution in a 1:1 (v/v) ratio to test for coacervate formation.

#### Brightfield Microscopy^16^

Microscope slides were prepared by affixing a 1.5 mm Fisherbrand coverslip to a Fisherbrand Superfrost slide using a double-stick tape at all four sides with a small channel left open to allow the sample to be drawn under the coverslip by capillary action. Each freshly prepared polypeptide coacervate sample was allowed to stand for 20 min to allow liquid coacervate droplets to condense or for precipitate to settle onto the slide surface. Samples were then imaged using a Zeiss Axio Observer Z1 optical Microscope. Microscope slides for imaging bulk coacervates isolated via centrifugation were prepared by spreading the sample on a Fisherbrand Superfrost slide and pipetting 50 μL of 150 mM PBS buffer on top to prevent evaporation.

#### Circular Dichroism Spectroscopy^16^

A sample of **Leu-*****rac*****-C**_**65**_ was analyzed at a concentration of 0.5 mg/mL in DI H_2_O adjusted to pH 7.0 with 0.1 M NaOH. The data were reported in units of molar ellipticity [θ] (deg·cm^2^·dmol^–1^), which was calculated using [θ] = (θ × 100 × MW)/(*c* × *l*), where θ is the measured ellipticity (millidegrees), MW is the average residue molecular mass (g/mol), *c* is the polypeptide concentration (mg/mL), and *l* is the cuvette path length (cm).

#### ζ-Potential Measurements^16^

Samples of **Xaa-*****rac*****-C**_**65**_, **Leu-C^H^**_**60**_, and **K**_**60**_ were each dissolved at 5 mg/mL in filtered (0.45 μm) DI H_2_O containing 20 mM NaCl. A coacervate suspension of **Met**^**O**^**-*****rac-*******C^O^**_**65**_** was prepared at 5 mg/mL in filtered (0.45 μm) DI H_2_O containing 150 mM NaCl and 12 mM TPP at pH 7.0. For polypeptides, samples were adjusted to pH 2.0 using filtered (0.45 μm) aqueous 1.0 M HCl. The samples were titrated to pH 12 with filtered (0.45 μm) aqueous 1.0 M NaOH. Aliquots (1 mL) were removed from the solutions over a range of pH values, and ζ-potential measurements of samples were acquired using a Zetasizer NanoZS instrument (Malvern Instruments Ltd., UK). The electrophoretic mobility was calculated in monomodal mode where only the fast field reversal (FFR) technique was applied to avoid degradation of the cuvette electrodes. Malvern Zetasizer Software was used to calculate electrophoretic mobility using the Henry equation. For the coacervate suspension of **Met**^**O**^**-*****rac-*******C^O^**_**65**_** prepared with 150 mM NaCl and 12 mM TPP at pH 7.0, the ζ-potential was found to be −4.4 ± 0.8 mV.

#### Cloud Point Temperature Measurements^16^

Samples of **Xaa-*****rac*****-C**_**65**_ or **Leu-*****rac*****-**C^O^**_**65**_** were prepared at 3.0 mg/mL in DI H_2_O containing a sodium salt of different counterions (12 mM) and NaCl (150 mM) or 1× PBS buffer (150 mM). Individual stock solutions of polypeptides (6.0 mg/mL) in DI H_2_O and stock solutions of sodium salts (120 mM), NaCl (2 M), and 10× PBS (1500 mM) at pH 5.0, 7.0, and 9.0 were used in sample preparation. Samples were prepared by mixing a polypeptide sample solution, a pH 7.0 stock solution of a sodium salt (phosphate, pyrophosphate, or tripolyphosphate), and NaCl solution in a 1:0.2:0.15 (v/v/v) ratio and then diluting with DI H_2_O to achieve a final polypeptide concentration of 3.0 mg/mL. The samples in PBS buffer were prepared by mixing a polypeptide sample solution and 2× pH 7.0 PBS buffer in a 1:1 (v/v) ratio to obtain a final polypeptide concentration of 3.0 mg/mL. The final pH of these samples was adjusted as desired by the addition of small volumes of the same polypeptide sample prepared using either pH 5.0 or 9.0 stock solutions. Transmittance of each sample at 500 nm was measured on a HP 8453 spectrophotometer equipped with an Agilent 8909A temperature controller. In initial runs, temperature was increased at a rate of 10 °C/min from 20 to 80 °C, followed by additional experiments within a selected temperature range at a heating rate of 2 °C/min. Absorbance data were converted to transmission and then normalized to span 0–100%. Cloud point temperatures (*T*_cp_) were determined as the temperature at 50% transmission.

#### Reversibility of Thermal Coacervation^16^

Studies of the reversibility of thermal coacervation were performed using samples **Leu-*****rac*****-C**_**65**_ and **Met-*****rac*****-C**_**65**_ at concentrations of 3.0 mg/mL in 1× PBS (150 mM) prepared as described above. The transmittance of each sample at 500 nm was measured on an HP 8453 spectrophotometer equipped with an Agilent 8909A temperature controller as described above. For each sample, the temperature was cycled at a rate of 3 °C/min from 20 to 50 °C, and this cycle was repeated 5 times ending at 20 °C.

#### Coacervate Formation as a Function of NaCl Concentration^16^

A stock solution of **Leu-*****rac*****-C**_**65**_ or **Met-*****rac*****-C**_**65**_ at 6.0 mg/mL in DI H_2_O was prepared and mixed in a 1:1 (v/v) ratio with separate stock solutions of TPP containing different concentrations of NaCl to give a series of samples with 3.0 mg/mL polypeptide and 12 mM TPP, with NaCl concentrations of 0, 0.05, 0.15, 0.25, 0.50, 1.0, and 2.0 M. Samples that formed coacervates were allowed to stand in 1.5 mL Eppendorf tubes for 24 h, followed by removal of the water-rich supernatant, which was then transferred into 2000 Da MWCO dialysis tubing. The samples were dialyzed against 1 M NaCl and 3 mM HCl (1×), 3 mM HCl (2×), and DI H_2_O (3×), with a minimum of 4 h between dialysate changes, before being lyophilized and weighed. This procedure converted all counterions to chloride for accurate quantification of the polypeptide. The fraction of **Leu-*****rac*****-C**_**65**_ or **Met-*****rac*****-C**_**65**_ in the coacervate phase for each sample was calculated by subtracting the mass of polypeptide recovered in the supernatant from the total mass of polypeptide initially added to the sample, which is divided by the total mass of polypeptide initially added to the sample. Mass fraction measurements were performed in triplicate.

## Results and Discussion

**Scheme 2:**
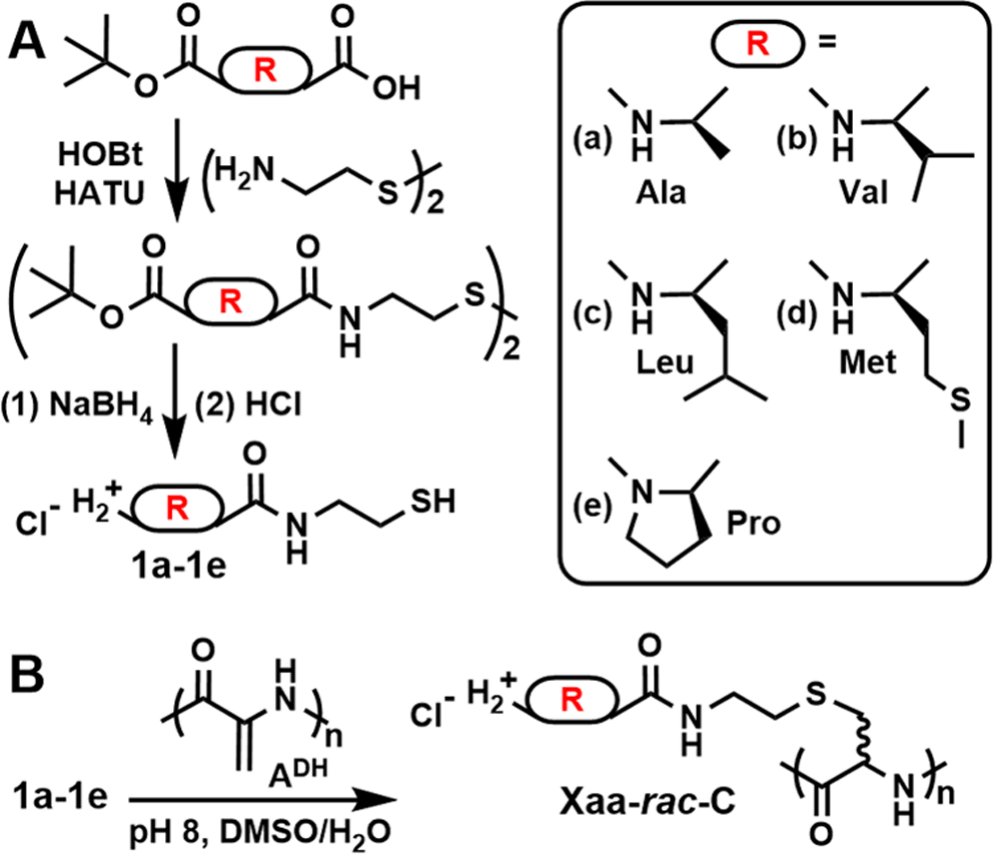
Synthesis of l-Amino Acid 2-Mercaptoethylamides **1a**–**1e** and Corresponding Polypeptides **Xaa-*****rac*****-C***_**n**_* Additional details are provided in the Experimental Section (see Supporting Information).

To obtain samples for comparison to previously reported **Xaa-C**^**H**^, a series of amino acid-functionalized poly(S-alkyl-*rac*-cysteines), **Xaa-*****rac*****-C***_**n**_*, were prepared as shown in Scheme 2. These samples all contain nonpolar amino acids and terminal primary α-amine groups in the side chains, which allow the formation of cationic polypeptides similar to **Xaa-C**^**H**^. A modular approach was used to prepare samples of **Xaa-*****rac*****-C**_**65**_ by using readily prepared L-amino acid 2-mercaptoethylamides **1a**–**1e**, (see Table S1), where the amino acids l-alanine (Ala), l-valine (Val), l-leucine (Leu), methionine (Met), and l-proline (Pro) were selected to provide a range of hydrophobicity. Using our recently developed methodology for preparation and modification of **A**^**DH**^,^17^ the free thiol groups in **1a**–**1e** were individually reacted with a common **A^DH^**_**65**_ precursor to give each **Xaa-*****rac*****-C**_**65**_ sample (overall isolated yields for complete polypeptide modification *ca*. 70–93%, Scheme 2 and Table S1). The chain length used was selected to enable comparison to previously published **Xaa-C**^**H**^ samples of similar length.^16^**Xaa-*****rac*****-C**_**65**_ was purified by dialysis against aqueous HCl (3 mM) followed by deionized water to give polypeptides as white powders after lyophilization. All of the isolated protonated polypeptides **Xaa-*****rac*****-C**_**65**_ as Cl^–^ salts were soluble in deionized water at 20 °C.

**A**^**DH**^ residues were fully converted into the corresponding functionalized residues for each **Xaa-*****rac*****-C**_**65**_ as determined by ^1^H and ^13^C NMR analyses, and we also found no evidence for chain degradation during modification similar to other previously reported reactions on **A**^**DH**^ (Figure S1).^17^ Circular dichroism analysis of **Leu-*****rac*****-C**_**65**_ in water gave no signal as expected for a racemic polypeptide (Figure S2).^17^ This result suggests that there is no chiral ordering of the homochiral side-chain groups, which also supports the adoption of disordered conformations. GPC analysis of **Xaa-*****rac*****-C**_**65**_ samples was accomplished using trifluoroethanol (TFE) containing sodium trifluoroacetate mobile phase. Under these conditions, all **Xaa-*****rac*****-C**_**65**_ samples gave monomodal distributions with some apparent high molecular weight tailing likely due to polypeptide aggregation (Figure S3). Chain lengths were also determined using end-group analysis (Table S1). Attempts to perform GPC in other solvents (DMF, hexafluoroisopropanol) or at higher concentrations in TFE showed increased levels of polypeptide aggregation. Molecular weights and dispersity values were found to be similar for all **Xaa-*****rac*****-C**_**65**_ samples analyzed in trifluoroethanol (Table S1), consistent with the expected structures. Overall, **Xaa-*****rac*****-C**_**65**_ were successfully prepared in good yields and high purity. A potential advantage of this process for preparation of **Xaa-*****rac*****-C** versus that for **Xaa-C**^**H**^ is the ability to directly conjugate a more diverse range of amino acids (e.g., Met) to the polypeptide backbone. In addition, the enhanced reactivity of **A**^**DH**^ with thiols versus amines eliminates the need to protect amine groups during conjugation.

**Figure 1:**
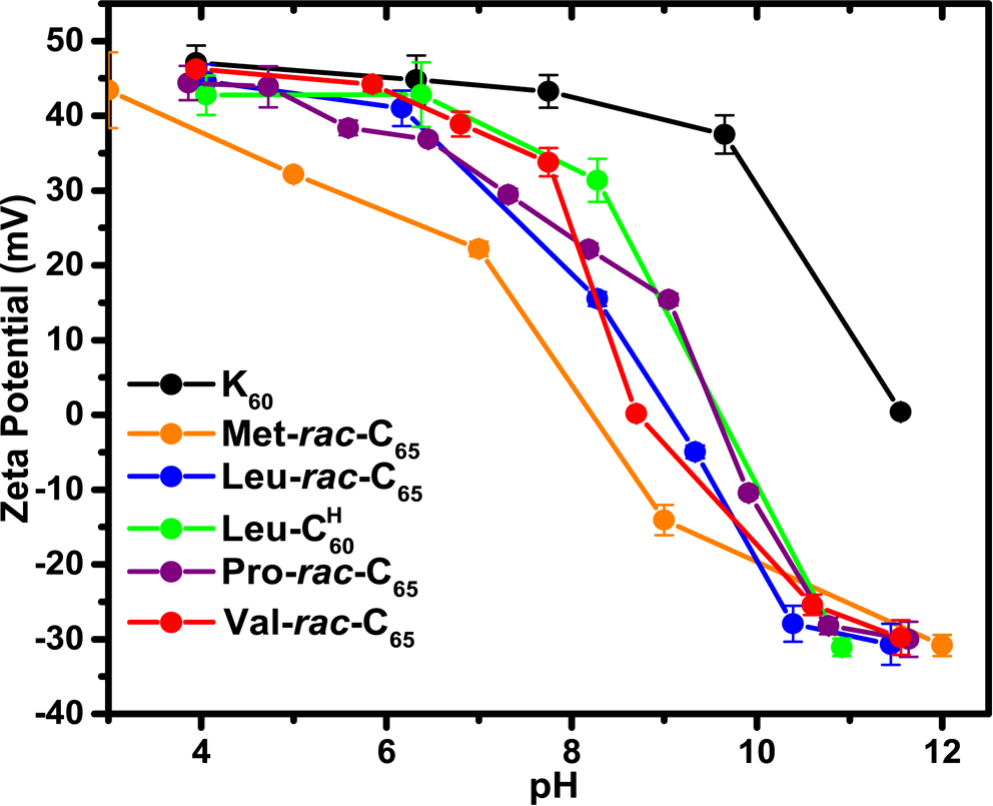
ζ-Potential measurements of polypeptide samples as a function of solution pH. Measurements of **Xaa-*****rac*****-C**_**65**_ and reference polypeptides were performed at 5.0 mg/mL in 20 mM NaCl at 20 °C. **Met-*****rac*****-C**_**65**_ = orange; **Leu-*****rac*****-C**_**65**_ = blue; **Pro-*****rac*****-C**_**65**_ = magenta; **Val-*****rac*****-C**_**65**_ = red; poly(l-lysine·HCl)_60_ (**K**_**60**_) = black; **Leu-C^H^**_**60**_ = green. Error bars represent standard deviations of triplicate measurements.

To compare the solution properties of **Xaa-*****rac*****-C**_**65**_ with those of **Xaa-C**^**H**^, we examined the degree of **Xaa-*****rac*****-C**_**65**_ protonation in 20 mM aqueous NaCl as a function of pH. Similar to results obtained with **Xaa-C**^**H**^,^16^ ζ-potentials for solutions of **Xaa-*****rac*****-C**_**65**_ measured at different pH revealed transitions from positive to moderately negative values between pH 7 and 10, which indicate a change in the α-amine groups from protonated to nonprotonated states over this pH range (Figure 1).^24^ This transition correlates with the expected p*K*_a_ range of α-amino amide groups (ca. 8)^25^ and was corroborated by analysis of other control samples that included **Leu-C^H^**_**60**_ and poly(l-lysine·HCl)_60_, **K**_**60**_, where the latter undergoes a charged to noncharged transition at a much higher pH (p*K*_a_ ca. 10) (Figure 1).^24,26^ It is noteworthy that the **Met-*****rac*****-C**_**65**_ sample was found to be partially protonated at lower pH compared to the other **Xaa-*****rac*****-C**_**65**_ samples, where the observed broader transition may reflect a wider range of p*K*_a_ values for this polypeptide. Overall, it was found that **Xaa-*****rac*****-C**_**65**_ can undergo protonation and deprotonation in water within a physiologically relevant range that is advantageous for adjustment of physical properties.

**Figure 2:**
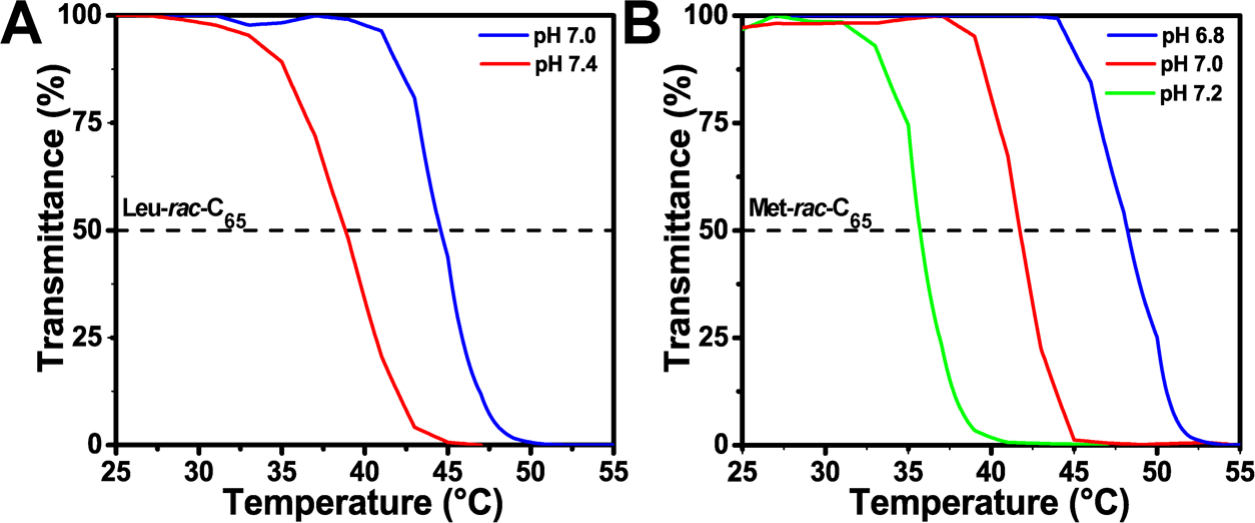
Temperature-dependent coacervate formation in solutions of (A) **Leu-*****rac*****-C**_**65**_ and (B) **Met-*****rac*****-C**_**65**_ as a function of pH. Panels show optical transmittance at 500 nm for 3.0 mg/mL solutions of polypeptides in 150 mM PBS buffer measured over a range of temperatures at different pH.

We evaluated physical properties by studying the phase behavior of **Xaa-*****rac*****-C**_**65**_ as functions of varying pH, temperature, and counterions in water. Initially, dilute aqueous solutions of **Xaa-*****rac*****-C**_**65**_ (3.0 mg/mL) were prepared in 150 mM phosphate buffer saline (PBS) buffer at pH values ranging from 6.8 to 8.0, which were chosen to include varying degrees of polypeptide protonation. These solutions were heated at a controlled rate up to 90 °C, and optical transmittance at 500 nm was measured as a function of temperature. The samples **Ala-*****rac*****-C**_**65**_ and **Pro-*****rac*****-C**_**65**_ were the least hydrophobic and were found to both remain soluble up to 90 °C (see Table 1). The **Val-*****rac*****-C**_**65**_, **Leu-*****rac*****-C**_**65**_, and **Met-*****rac*****-C**_**65**_ samples are more hydrophobic and were found to phase separate by variation of both pH and temperature (Figures 2 and S4 and Table 1). Reversible cloud points were observed for the Val, Leu, and Met samples upon increase in temperature, indicative of lower critical solution temperature (LCST) induced phase separation,^27^ with cloud point temperatures (*T*_cp_) that decreased with increasing hydrophobicity of side-chain amino acids in the approximate order Val < Leu ∼ Met (Table 1).^28^ Cloud point temperatures for these samples also decreased as pH was increased, which is consistent with the properties previously observed with **Xaa-C**^**H**^.^16^ These results confirm that **Val-*****rac*****-C**_**65**_, **Leu-*****rac*****-C**_**65**_, and **Met-*****rac*****-C**_**65**_ in 150 mM PBS buffer are able to phase separate by variation of temperature, ionic strength, and solution pH within a physiologically relevant range. It was also possible to tune *T*_cp_ in a predictable manner by variation of polypeptide side-chain amino acid hydrophobicity.

**Table 1:** Coacervate Transition Temperatures for Aqueous Solutions of **Xaa-rac-C**_**65**_ Containing Different Counterions^a^

		Xaa-rac-C_65_*T*_cp_ (°C)				
counterion	ion valency at pH 7.0	Ala	Val	Leu	Met	Pro
chloride	–1.0	sol	sol	sol	sol	sol
phosphate	–1.4	sol	71	45	41	sol
pyrophosphate	–2.7	×	×	–	–	×
tripolyphosphate	–4.0	ppt	–	–	–	ppt

a Cloud point temperatures (*T*_cp_) were measured for 3.0 mg/mL solutions of **Xaa-rac-C**_**65**_ in the presence of different counterions (12 mM) in 150 mM NaCl at pH 7.0. sol = polypeptide was soluble up to 90 °C; – = polypeptide formed a coacervate at 20 °C; × = not performed; ppt = polypeptide formed a solid precipitate, not a coacervate, at 20 °C.

Since we observed that the substitution of phosphate for chloride decreased the solubility of **Xaa-*****rac*****-C**_**65**_ in water, the effects of counterion valency on aqueous phase separation were further investigated. Aqueous samples of **Xaa-*****rac*****-C**_**65**_ (3.0 mg/mL) were prepared in 150 mM NaCl at pH 7.0 and then mixed with sodium salts of anionic counterions with different valencies (12 mM final concentration, Table 1). Similar to results observed with **Xaa-C**^**H**^,^16^ as anion valency was increased, all samples became less soluble in water. This result is explained by increased stability of ion complexes between the cationic polypeptides and anions with higher valency. Even the more hydrophilic **Ala-*****rac*****-C**_**65**_ and **Pro-*****rac*****-C**_**65**_ samples were found to undergo phase separation in the presence of tripolyphosphate anions at 20 °C. Since they both contain the same side-chain amino acid functionality, comparison of the **Leu-*****rac*****-C**_**65**_ and **Leu-C^H^**_**60**_ samples can provide some insight into how the different polypeptide backbones affect phase separation properties. In the presence of counterions of different valencies, **Leu-*****rac*****-C**_**65**_ possesses a lower *T*_cp_ compared **Leu-C^H^**_**60**_ under otherwise identical conditions (e.g., *T*_cp_ = <20 °C versus 57 °C with pyrophosphate, respectively).^16^ This trend suggests that disordered ***rac*****-C** chains provide a more hydrophobic local environment compared to the α-helical **C**^**H**^ chains, which may also be partially explained by the presence of polar hydroxyl groups in the side-chains of **Xaa-C^H^**_**60**_ polypeptides.

**Figure 3:**
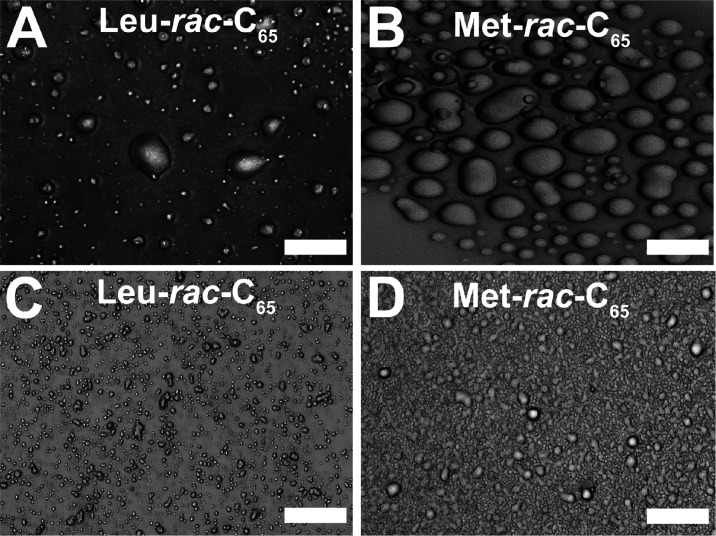
Optical micrographs of **Leu-*****rac*****-C**_**65**_ and **Met-*****rac*****-C**_**65**_ mixed with sodium tripolyphosphate (TPP) or polyadenylic acid (polyA). Solutions of either **Leu-*****rac*****-C**_**65**_ or **Met-*****rac*****-C**_**65**_ at 3.0 mg/mL in 150 mM NaCl at 20 °C and pH 7.0 were mixed with TPP (12 mM final concentration) or at 5.0 mg/mL in 150 mM NaCl at 20 °C and pH 7.0 with polyA (0.015 mM final concentration). The resulting turbid suspensions were allowed to settle onto glass slides before imaging. (A) **Leu-*****rac*****-C**_**65**_ + TPP; (B) **Met-*****rac*****-C**_**65**_ + TPP; (C) **Leu-*****rac*****-C**_**65**_ + polyA; (D) **Met-*****rac*****-C**_**65**_ + polyA. Scale bars = 20 μm.

**Figure 4:**
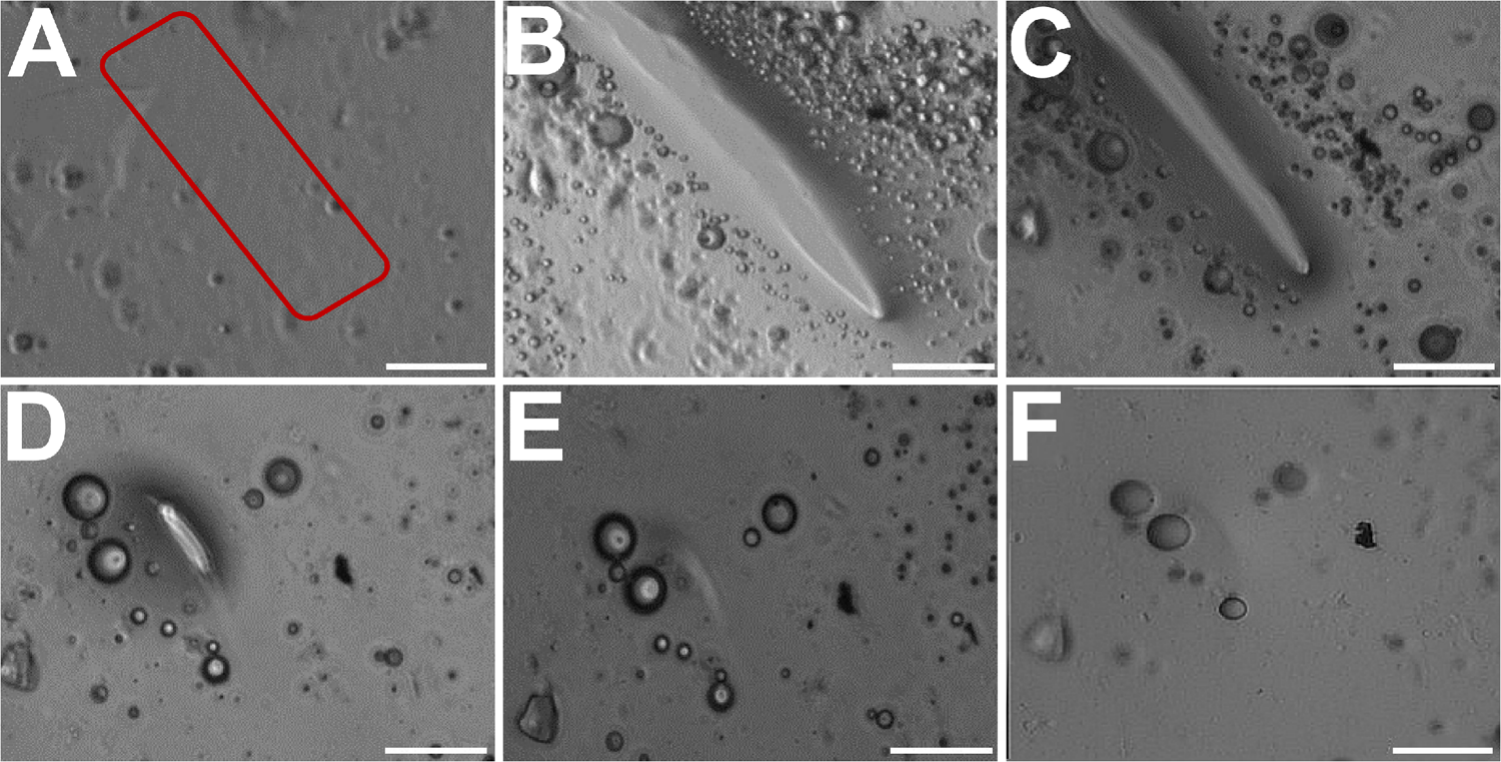
Viscous liquid properties observed over time in optical micrographs of a coacervate film prepared by mixing **Met-*****rac*****-C**_**65**_ at 3.0 mg/mL with TPP in 150 mM NaCl at 20 °C and pH 7.0. (A) The coacervate was collected by centrifugation and then spread as a film on a glass slide. PBS buffer (150 mM, pH 7.0) was placed on top of the film to prevent evaporation, and a defect was made by scraping the film with a needle (red box area). (B) Image of the film 5 min after defect was made. (C) Image of the film after 10 min; note decrease in defect size due to flow of coacervate. (D) Image of the film after 20 min. (E) Image of the film after 25 min. (F) Image of the film after 30 min; note defect has completely healed. Scale bars = 20 μm.

To investigate the nature of phase separation described above for **Xaa-*****rac*****-C**_**65**_ samples, the suspensions formed in the presence of tripolyphosphate anions at pH 7.0 were allowed to settle onto glass slides and were examined using optical microscopy. Images of the complexes formed with least hydrophobic **Ala-*****rac*****-C**_**65**_ and **Pro-*****rac*****-C**_**65**_ samples showed irregular edges and clusters consistent with the formation of solid precipitates (Figure S5). However, images of tripolyphosphate complexes with **Val-*****rac*****-C**_**65**_, **Leu-*****rac*****-C**_**65**_, and **Met-*****rac*****-C**_**65**_ all showed smooth outlines and coalescing droplets consistent with the formation of liquid coacervate phases (Figures 3a,b and S5).^5,6^ For additional confirmation of coacervate formation, centrifugation of **Met-*****rac*****-C**_**65**_ with tripolyphosphate suspension resulted in the formation of two separate liquid layers (see Figure S5). A thin film of the coacervate of **Met-*****rac*****-C**_**65**_ with tripolyphosphate was also spread as a thin film on a glass slide. A defect made in the film was observed to heal by the flow of coacervate within ca. 30 min, which confirmed that the sample possessed viscous liquid properties (Figure 4). In addition to coacervation with tripolyphosphate, **Leu-*****rac*****-C**_**65**_ and **Met-*****rac*****-C**_**65**_ were also able to form complex coacervates with a model RNA sequence, polyadenylic acid, at 20 °C (Figure 3c,d). These results are comparable to those found with **Xaa-C**^**H**^ samples and suggest that the combination of hydrophobic and charged amino acid groups in side chains is a robust means to obtain coacervate-forming polypeptides.

**Figure 5:**
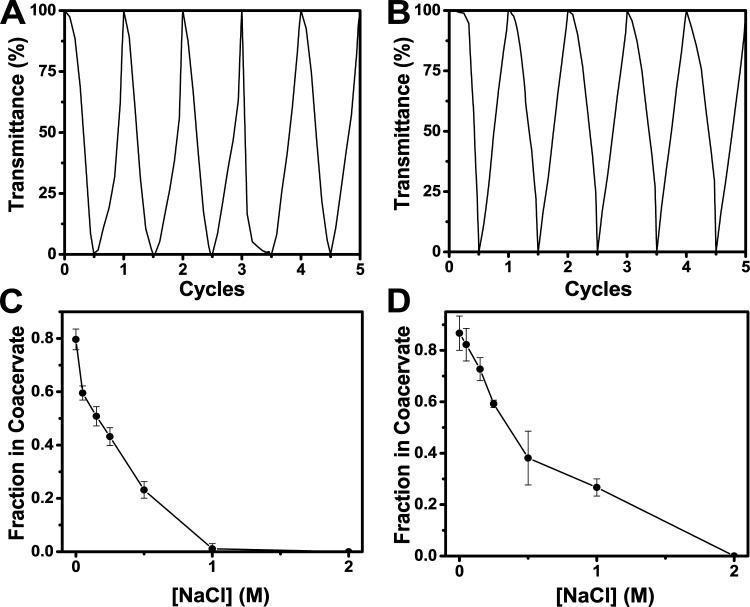
Reversibility of temperature-dependent coacervate formation for (A) **Leu-*****rac*****-C**_**65**_ and (B) **Met-*****rac*****-C**_**65**_ in PBS buffer as determined by optical transmittance at 500 nm. Samples were prepared at 3.0 mg/mL in 150 mM PBS buffer at pH 7.2 and subjected to repeated thermal cycling between 25 and 40 °C. Samples were heated and cooled at the rate of 3 °C/min. Coacervate formation of (C) **Leu-*****rac*****-C**_**65**_ and (D) **Met-*****rac*****-C**_**65**_ both mixed with TPP as a function of sodium chloride concentration. Fraction in Coacervate = (mass of polypeptide in coacervate phase)/(total mass of polypeptide). Samples were prepared by mixing each polypeptide (3.0 mg/mL) in media with TPP (12 mM) and different concentrations of NaCl at 20 °C and pH 7.0. Error bars represent standard deviations of triplicate measurements.

Coacervate formation with **Leu-*****rac*****-C**_**65**_ and **Met-*****rac*****-C**_**65**_ was found to be reversible with no irreversible formation of polypeptide aggregates. Multiple heating and cooling cycles were applied to samples of **Leu-*****rac*****-C**_**65**_ and **Met-*****rac*****-C**_**65**_ in PBS buffer at pH 7.2 and monitored by optical transmittance, where coacervation and complete dissolution were observed with each thermal cycle (Figure 5a,b). Finally, to assess salt stability, complexes of **Leu-*****rac*****-C**_**65**_ and **Met-*****rac*****-C**_**65**_ with tripolyphosphate were prepared in aqueous solutions containing 0.1 to 2 M NaCl. The fraction of each polypeptide that partitioned into the coacervate phase decreased with increasing ionic strength, and phase separation did not occur at higher salt concentrations (Figure 5c,d). The **Leu-*****rac*****-C**_**65**_ and **Met-*****rac*****-C**_**65**_ coacervates were found to be significantly more stable at higher ionic strength (stable to 1 and 2 M NaCl, respectively) compared to the previously reported **Leu-C^H^**_**60**_ sample (stable to 250 mM NaCl) under otherwise identical conditions.^16^ The increased stability of the **Xaa-*****rac*****-C**_**65**_ samples is likely due to their increased hydrophobicity compared to **Xaa-C**^**H**^ samples, as noted above. The increased salt stability of **Met-*****rac*****-C**_**65**_ coacervates may be beneficial for downstream applications.

**Scheme 3:**
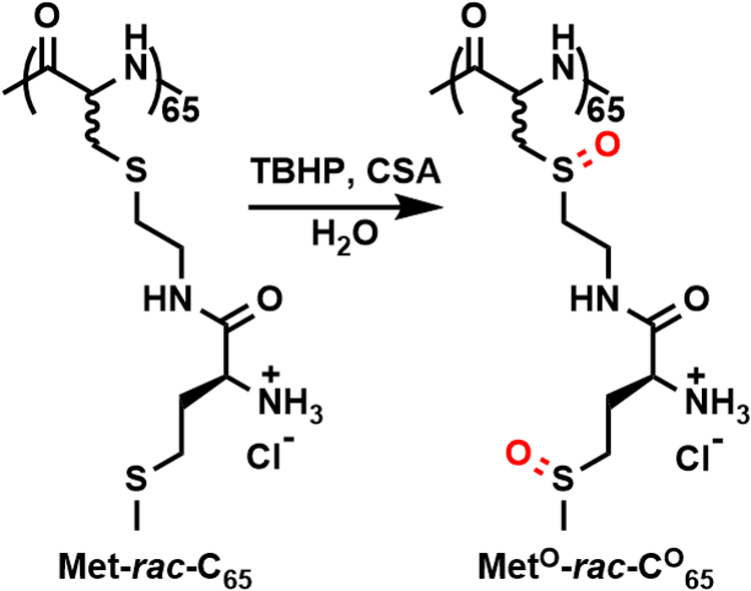
Synthesis of Sulfoxide Derivative **Met**^**O**^**-*****rac*****-**C^O^**_**65**_** from **Met-*****rac*****-C**_**65**_ TBHP = *tert*-butyl hydroperoxide; CSA = camphorsulfonic acid.

We had previously shown that the thioether linkages in **Leu-C^H^**_**60**_ could be oxidized under mild conditions, which resulted in the disruption of its α-helical conformation as well as dissolution of its coacervate with tripolyphosphate due to increased hydrophilicity of the resulting sulfoxide groups.^16^ Since thioether oxidation provides a mild means to actively control coacervation under physiologically relevant conditions, we sought to evaluate its effect on coacervates of **Leu-*****rac*****-C**_**65**_ and **Met-*****rac*****-C**_**65**_. Samples of **Leu-*****rac*****-C**_**65**_ and **Met-*****rac*****-C**_**65**_ were oxidized to give the corresponding sulfoxide derivatives **Leu-*****rac*****-**C^O^**_**65**_** and **Met**^**O**^**-*****rac*****-**C^O^**_**65**_** using *tert*-butyl hydroperoxide in water (Schemes 3 and S1).^24^ The increased hydrophilicity of **Leu-*****rac*****-**C^O^**_**65**_** and **Met**^**O**^**-*****rac*****-**C^O^**_**65**_** relative to the unoxidized precursors had a striking effect on their ability to form coacervates. Mixtures of aqueous solutions of **Leu-*****rac*****-**C^O^**_**65**_** and **Met**^**O**^**-*****rac*****-**C^O^**_**65**_** with tripolyphosphate anions at pH 7.0 and 20 °C did not form coacervates and appeared to remain as transparent solutions. However, heating of the **Leu-*****rac*****-**C^O^**_**65**_** sample containing tripolyphosphate was able to induce coacervate formation at *ca*. 33 °C. Alternatively, raising the pH of this mixture to 8.5 at 20 °C also resulted in coacervate formation (Figure S6). Mixture of an aqueous solution of **Leu-*****rac*****-**C^O^**_**65**_** with polyadenylic acid at pH 7.0 and 20 °C also resulted in coacervate formation (Figure S7). These results show that **Leu-*****rac*****-**C^O^**_**65**_** is more hydrophilic than its unoxidized precursor **Leu-*****rac*****-C**_**65**_ but can still form coacervates under certain conditions.

On the contrary, the sample of **Met**^**O**^**-*****rac*****-**C^O^**_**65**_** containing tripolyphosphate at pH 7.0 did not phase separate even up to 90 °C. The mixture of an aqueous solution of **Met**^**O**^**-*****rac*****-**C^O^**_**65**_** with polyadenylic acid at pH 7.0 and 20 °C also resulted in no visible phase separation. To study these samples in more detail, the mixtures of **Met**^**O**^**-*****rac*****-**C^O^**_**65**_** with tripolyphosphate and polyadenylic acid were analyzed using dynamic light scattering, which revealed the presence of coacervate nanodroplets (Figure S8). Solutions of the individual components showed negligible aggregation on their own, which supports the formation of polyion complexes in the mixtures (Figure S9). Measurement of ζ-potential on the nanodroplets formed with **Met**^**O**^**-*****rac*****-**C^O^**_**65**_** containing tripolyphosphate at pH 7.0 gave a value of −4.4 ± 0.8 mV, which may explain the colloidal stability of the droplets being due to incorporation of excess tripolyphosphate anions. Greater hydrophilicity of **Met**^**O**^**-*****rac*****-**C^O^**_**65**_** compared to **Leu-*****rac*****-**C^O^**_**65**_** may also explain the decreased ability of **Met**^**O**^**-*****rac*****-**C^O^**_**65**_** to form macroscopic coacervates. We hypothesize that the oxidation of two thioether groups per residue in **Met-*****rac*****-C**_**65**_ allows for a greater range of polarity switching compared to **Leu-*****rac*****-C**_**65**_ that has only a single thioether group per residue. The extra thioether groups in **Met-*****rac*****-C**_**65**_ allow for switching between highly hydrophobic and highly hydrophilic states via oxidation.

**Figure 6:**
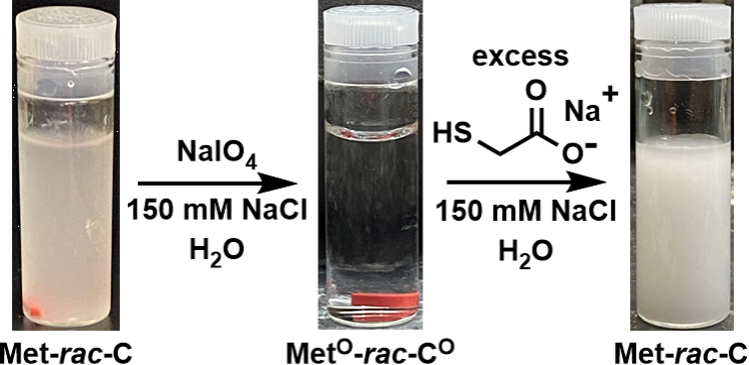
Reversible oxidation and reduction of a **Met-*****rac*****-C**_**65**_ and TPP coacervate. Coacervate suspension (left) was formed by mixing **Met-*****rac*****-C**_**65**_ at 5.0 mg/mL with TPP (13 mM) in 150 mM NaCl at 20 °C and pH 7.0. The coacervate was cooled to 0 °C and then oxidized by the addition of 1.1 equiv of NaIO_4_ to give a transparent mixture of **Met**^**O**^**-*****rac*****-**C^O^**_**65**_** and TPP (middle). Excess NaIO_4_ was then removed from the sample by dialysis against TPP (13 mM) in 150 mM NaCl at 20 °C and pH 7.0. Adjustment of this **Met**^**O**^**-*****rac*****-**C^O^**_**65**_** sample pH to 8.5 with 0.1 M NaOH followed by the addition of excess thioglycolic acid, giving a pH of 5 to 6, resulted in a complete reduction to **Met-*****rac*****-C**_**65**_. Reformation of the coacervate suspension (right) occurred when pH was increased to 7.0 using 12 M NaOH after reduction was complete.

To further examine the redox-mediated coacervate switching properties of **Met-*****rac*****-C**_**65**_, we sought to demonstrate *in situ* oxidation and reduction of a coacervate of **Met-*****rac*****-C**_**65**_ and tripolyphosphate. Slightly more than 1 equiv of NaIO_4_ oxidant was added to a coacervate suspension of **Met-*****rac*****-C**_**65**_ with tripolyphosphate in NaCl (150 mM) at 0 °C and pH 7.0 (Figure 6). Within 5 min, the coacervate droplets had completely dissolved as **Met-*****rac*****-C**_**65**_ was oxidized to **Met**^**O**^**-*****rac*****-**C^O^**_**65**_**. After 2 h, excess NaIO_4_ was removed by brief dialysis against water containing tripolyphosphate and NaCl. The sample was then adjusted to pH 5 to 6 by addition of NaOH and excess thioglycolic acid, which resulted in complete reduction back to **Met-*****rac*****-C**_**65**_ and consequent reformation of the coacervate suspension after pH was increased to 7.0 (Figure 6). ^1^H NMR spectroscopy of aliquots of oxidized and reduced samples gave spectra identical to those of purified **Met**^**O**^**-*****rac*****-**C^O^**_**65**_** and **Met-*****rac*****-C**_**65**_ (see Supporting Information), indicating that both reactions went to completion. While we had reported similar coacervate switching of **Leu-C**_**60**_^**H**^ and tripolyphosphate,^16^ it was difficult to achieve a complete reduction in that system due to phase separation during the reaction. The wider p*K*_a_ range of **Met-*****rac*****-C**_**65**_ and greater aqueous solubility of **Met**^**O**^**-*****rac*****-**C^O^**_**65**_** compared to the **Leu-C^H^**_**60**_ system appear to provide an advantage in facilitating the redox reactions under mild conditions. These properties result in robust actively controlled polypeptide coacervation with **Met-*****rac*****-C**_**65**_ that utilizes nondestructive, physiologically compatible oxidation and reduction conditions. For example, the intracellular oxidation of methionine residues in proteins is well-known,^29^ and intracellular thiols and ubiquitous reductase enzymes can readily reduce methionine sulfoxide residues in proteins.^30−32^

## Conclusions

Building off of prior results on **Xaa-C**^**H**^ polypeptides that form coacervates in water,^16^ we prepared a new class of side-chain amino acid-functionalized **Xaa-*****rac*****-C** via post-polymerization modification of **A**^**DH**^.^17^ The **A**^**DH**^ platform allowed straightforward incorporation of a wider range of side-chain amino acid functional groups in **Xaa-*****rac*****-C** compared to the **Xaa-C**^**H**^ platform and also allowed use of fewer protecting groups. We found that **Xaa-*****rac*****-C** can also form coacervates with properties similar to those seen with **Xaa-C**^**H**^, where side-chain amino acid hydrophobicity can be used to alter coacervation properties, specifically the ability to respond to physiologically relevant environmental changes in pH, temperature, and counterions. These results suggest that incorporation of side-chain amino acids onto polypeptides may be a general way to obtain coacervation. Coacervates formed using **Met-*****rac*****-C** were found to possess improved stability against high ionic strength media. Further, the presence of an additional thioether group in each **Met-*****rac*****-C** side chain resulted in an increased solubility change upon oxidation allowing facile reversible redox switching of coacervate formation in aqueous media. These **Xaa-*****rac*****-C** polypeptides may be amenable for use as mimics of coacervate-forming proteins and in applications such as therapeutic delivery and protein separation.

## Supporting Information Available

Additional experimental procedures and data, additional figures, schemes, tables, and spectral data (PDF)
