# Supplementary material for: Switchable Coacervate Formation via Amino Acid Functionalization of Poly(dehydroalanine)
Source: Biomacromolecules. 2024 Mar 1;25(4):2554–62. doi: 10.1021/acs.biomac.4c00048 (PMC11005011; doi:10.1021/acs.biomac.4c00048)
Supplement: Supplementary file 1 — bm4c00048_si_001.pdf [file bm4c00048_si_001.pdf]

Supporting Information

Switchable coacervate formation via amino-acid functionalization of poly(dehydroalanine)

Casey A. Morrison[a], Ethan P. Chan[a], Thatcher Lee[b,c], Timothy J. Deming[a,c]*

[a] Department of Chemistry and Biochemistry, University of California, Los Angeles, CA 90095 USA
[b] Department of Chemistry, Smith College, Northampton, MA 01063 USA
[c] Department of Bioengineering, University of California, Los Angeles, CA 90095 USA

Table S1. Isolated yields of L-amino acid 2-mercaptoethylamides (**1a-1e**) and isolated yields and molecular weight properties of $\mathbf{C^{BCM}_{65}}$ and **Xaa-*rac*-C₆₅** polypeptides. All polypeptides had average degrees of polymerization ranging from 62 to 68 by ^{1}H NMR end-group analysis. a = Yields are total isolated yield from starting amino acid (3 steps). b = M_n and M_w for **Xaa-*rac*-C_{65}** polypeptides were calculated relative to PMMA standards in NaTFA/TFE; M_n and M_w for $\mathbf{C^{BCM}_{65}}$ was calculated relative to PEG standards in KTFA/HFIP. $Đ$ = M_w/M_n. N/A = not applicable.

Sample	Yield 1a-1e (%)[a]	Yield Polymer (%)	M_n[b]	M_w[b]	$Đ$[c]
$\mathbf{C^{BCM}_{65}}$	N/A	92	5,000	6,940	1.38
**Ala-*rac*-C_{65}**	65	71	11,040	14,600	1.32
**Val-*rac*-C_{65}**	83	70	10,330	13,240	1.28
**Leu-*rac*-C_{65}**	73	81	9,320	14,020	1.50
**Met-*rac*-C_{65}**	68	93	10,230	12,960	1.27
**Pro-*rac*-C_{65}**	74	61	10,280	14,310	1.39

Scheme S1. Synthesis of sulfoxide derivative **Leu-*rac*-C^{O}_{65}** from **Leu-*rac*-C_{65}**. TBHP = *tert*-butyl hydroperoxide. CSA = camphorsulfonic acid.

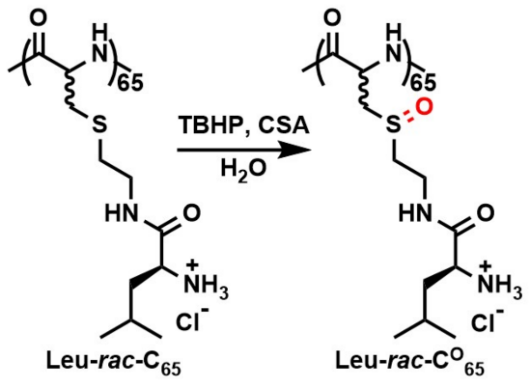

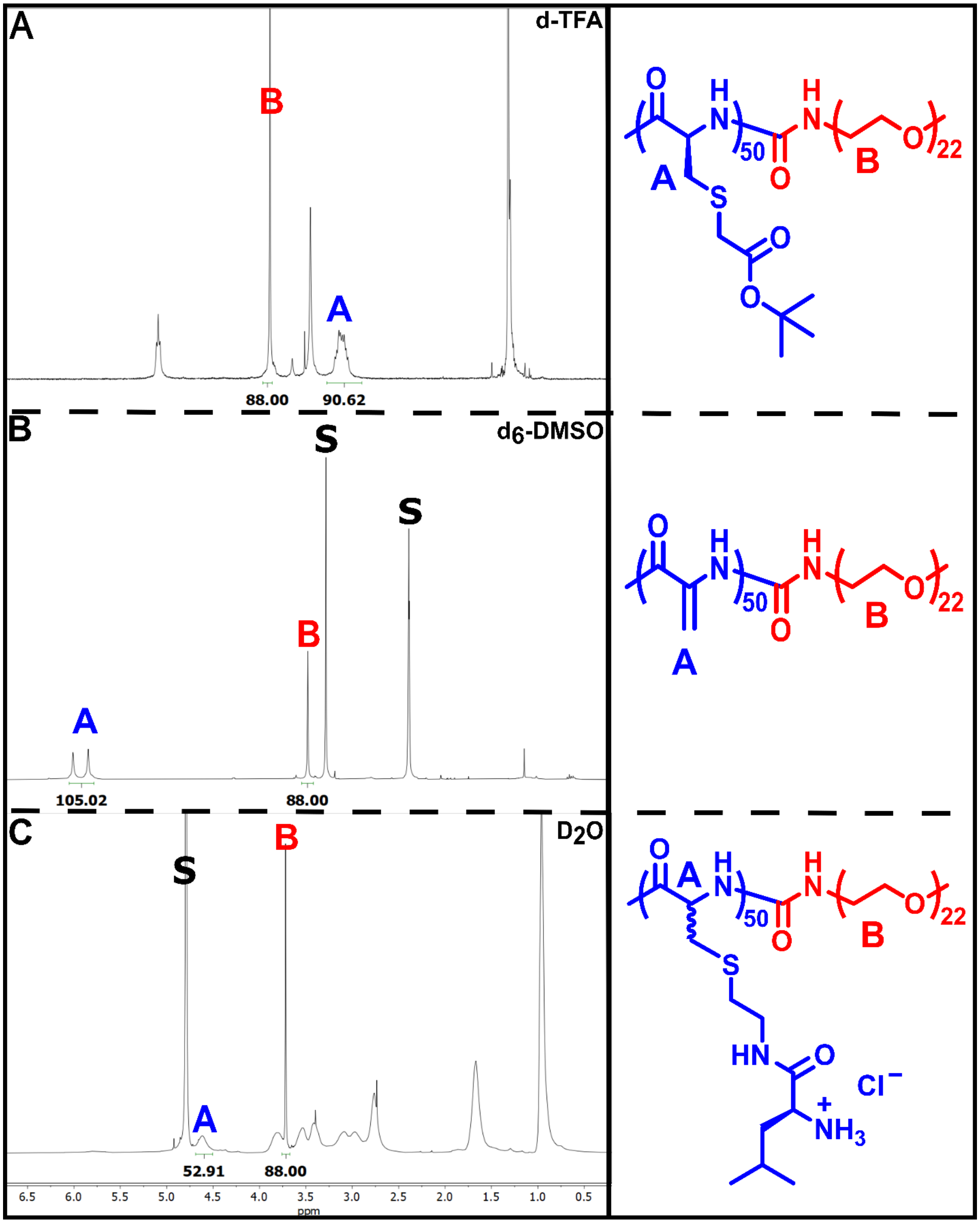

Figure S1. ^{1}H NMR spectra of pegylated polypeptide intermediates in the synthesis of **Leu-*rac*-C** showing negligible polypeptide chain cleavage during modifications. ^{1}H NMR spectra and structures of A) **PEG$_{22}$-*b*-C^{BCM}$_{50}$**, B) **PEG$_{22}$-*b*-A^{DH}$_{50}$**, and C) **PEG$_{22}$-*b*-(Leu-*rac*-C)$_{50}$**. Solvents are noted in upper right of each spectrum. S = solvent resonances. Degree of polymerization of the polypeptide segments was designated as an average value of 50.

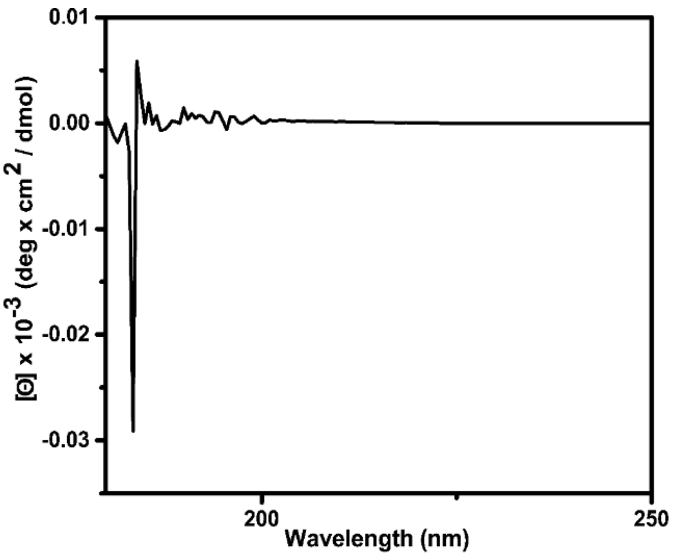

Figure S2. Circular dichroism spectrum of **Leu-*rac*-C$_{65}$** (0.5 mg/mL) in DI water adjusted to pH 7.0 with 0.1 M NaOH.

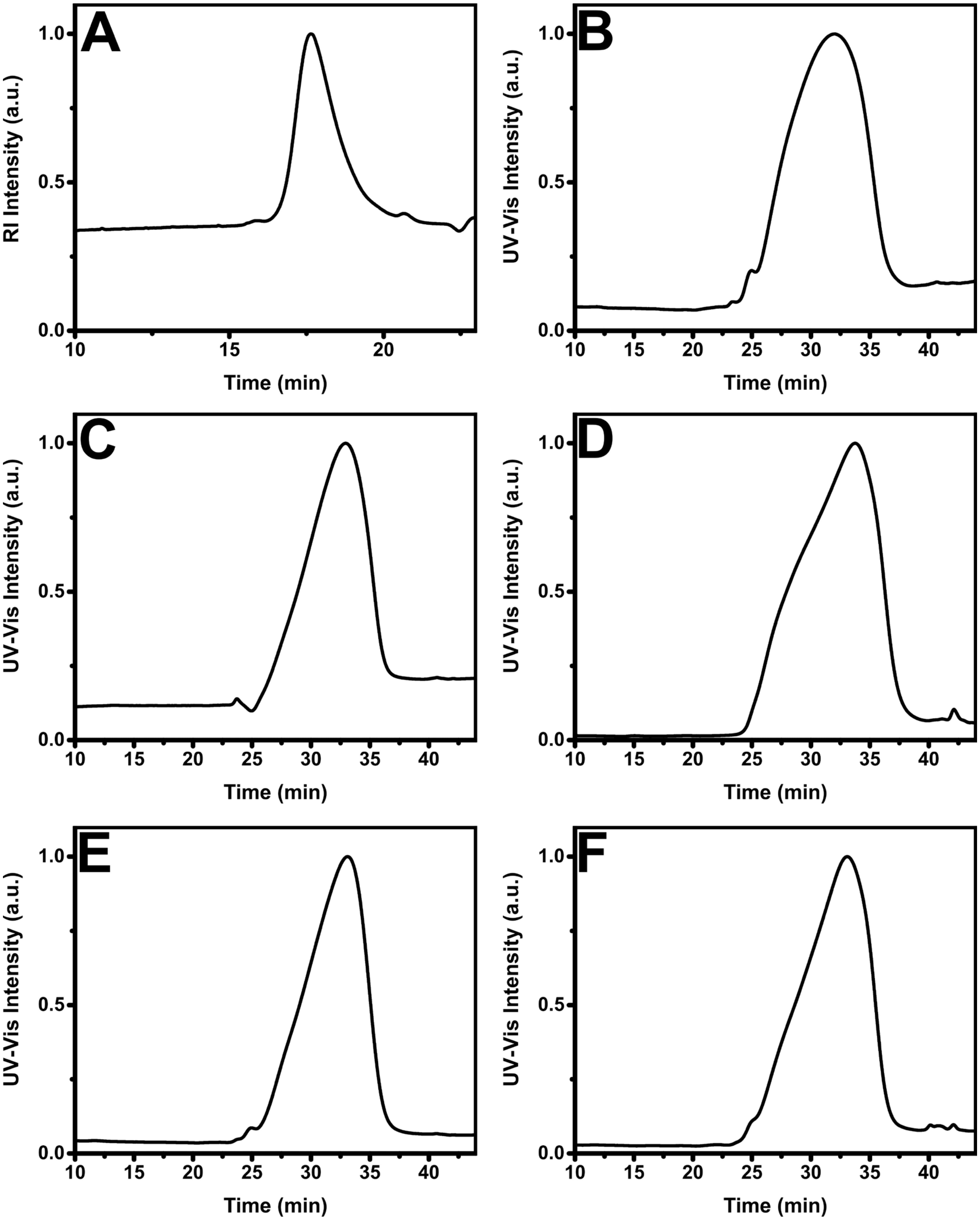

Figure S3. GPC analysis of polypeptides. A) $\mathbf{C^{BCM}_{65}}$ (20 mg/mL) in HFIP with 0.5% (w/v) KTFA at 40 °C. B) **Ala-*rac*-C$_{65}$** (0.5 mg/mL) in TFE with 20 mM NaTFA. C) **Val-*rac*-C$_{65}$** (0.5 mg/mL) in TFE with 20 mM NaTFA. D) **Leu-*rac*-C$_{65}$** (1.0 mg/mL) in TFE with 20 mM NaTFA. E) **Met-*rac*-C$_{65}$** (0.5 mg/mL) in TFE with 20 mM NaTFA. F) **Pro-*rac*-C$_{65}$** (0.5 mg/mL) in TFE with 20 mM NaTFA. All GPC analyses in TFE were at 25 °C.

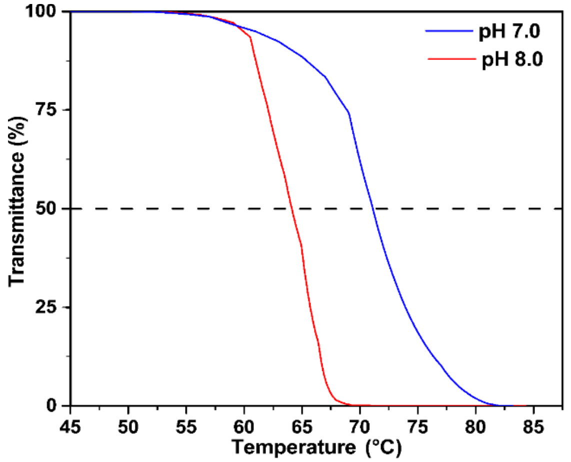

Figure S4. Temperature dependent coacervate formation in 3.0 mg/mL solutions of **Val-*rac*-C65** as a function of pH. Panel shows optical transmittance at 500 nm for 3.0 mg/mL solutions of **Val-*rac*-C65** in 150 mM PBS buffer measured over a range of temperature at different pH.

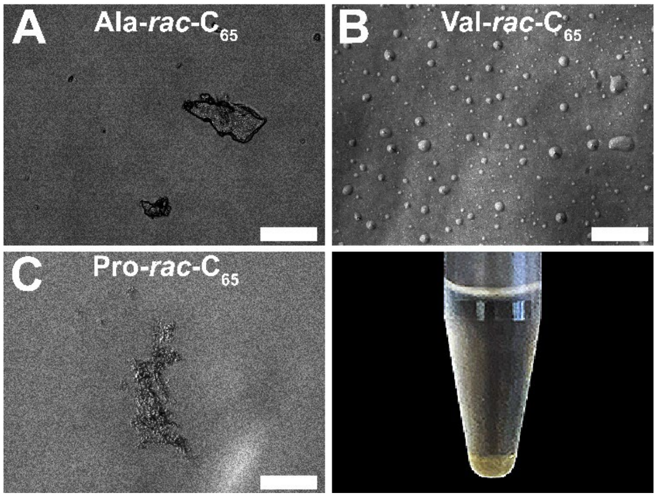

Figure S5. Optical micrographs of **Xaa-*rac*-C65** polypeptides mixed with sodium tripolyphosphate (TPP). Solutions of **Xaa-*rac*-C65** at 3.0 mg/mL in 150 mM NaCl at 20 °C and pH 7.0 were mixed with TPP (12 mM final concentration) and the resulting turbid suspensions were allowed to settle onto glass slides before imaging. A) **Ala-*rac*-C65** + TPP; B) **Val-*rac*-C65** + TPP; C) **Pro-*rac*-C65** + TPP. Scale bars = 20 µm. D) Image of **Met-*rac*-C65** + TPP coacervate (3.0 mg/mL in 150 mM NaCl at 20 °C and pH 7.0) after centrifugation. Condensed coacervate bulk phase can be seen at the bottom of the tube.

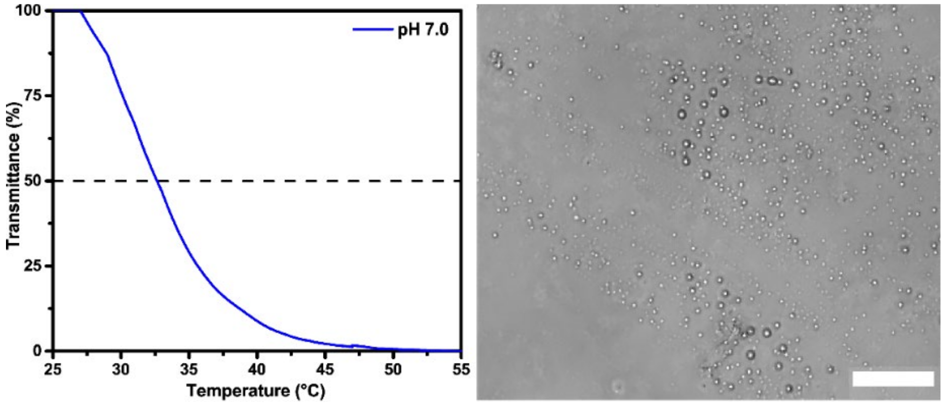

Figure S6. A) Temperature dependent coacervate formation in a 3.0 mg/mL solution of **Leu-*rac*-C$^{O}_{65}$** at pH 7.0. Plot shows optical transmittance at 500 nm for a 3.0 mg/mL solution of **Leu-*rac*-C$^{O}_{65}$** in 150 mM PBS buffer measured over a range of temperature at pH 7.0. B) Optical micrograph of **Leu-*rac*-C$^{O}_{65}$** mixed with TPP. A solution of **Leu-*rac*-C$^{O}_{65}$** at 3.0 mg/mL in 150 mM NaCl at 20 °C and pH 8.5 was mixed with TPP (12 mM final concentration) and the resulting turbid suspension was allowed to settle onto a glass slide before imaging. Scale bar = 20 μm.

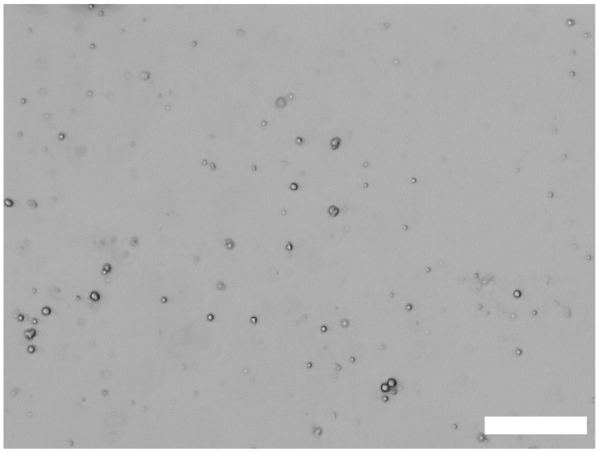

Figure S7. Optical micrograph of **Leu-*rac*-C$^{O}_{65}$** mixed with polyA. A solution of **Leu-*rac*-C$^{O}_{65}$** at 5.0 mg/mL in 150 mM NaCl at 20 °C and pH 7.0 was mixed with polyA (0.015 mM final concentration) and the resulting turbid suspension was allowed to settle onto a glass slide before imaging. Scale bar = 20 μm.

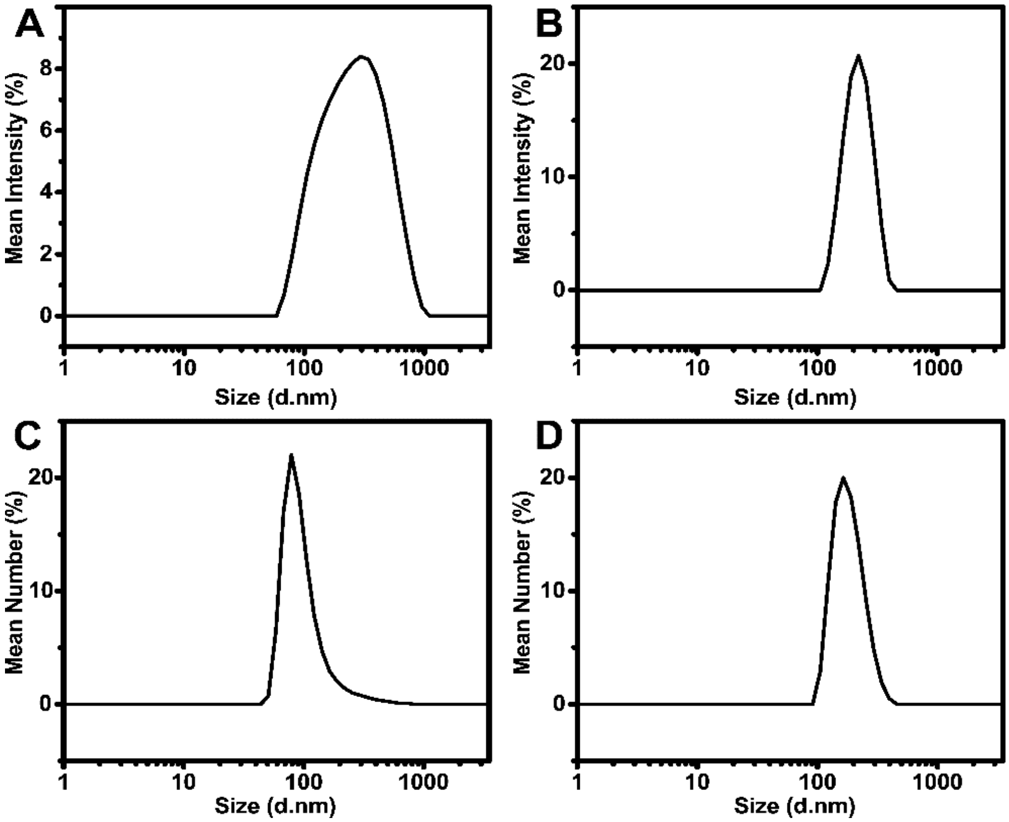

Figure S8. Size distributions from dynamic light scattering analysis of polypeptide samples over time. Intensity size distributions of samples of (A) 3.0 mg/mL **Met^{O}-*rac*-C^{O}$_{65}$** prepared in 150 mM NaCl at pH 7.0 with 12 mM TPP, and (B) 5.0 mg/mL **Met^{O}-*rac*-C^{O}$_{65}$** prepared in 150 mM NaCl at pH 7.0 with 0.015 mM polyA. Number size distributions of samples of (C) 3.0 mg/mL **Met^{O}-*rac*-C^{O}$_{65}$** prepared in 150 mM NaCl at pH 7.0 with 12 mM TPP, and (D) 5.0 mg/mL **Met^{O}-*rac*-C^{O}$_{65}$** prepared in 150 mM NaCl at pH 7.0 with 0.015 mM polyA. All samples were diluted to 0.1 mg/mL of **Met^{O}-*rac*-C^{O}$_{65}$** by addition of aqueous 150 mM NaCl at pH 7.0, and passed through a 0.45 μm pore size PTFE syringe filter before analysis using a Malvern Zetasizer Nano ZS at 20 °C. d.nm = average hydrodynamic diameter in nanometers.

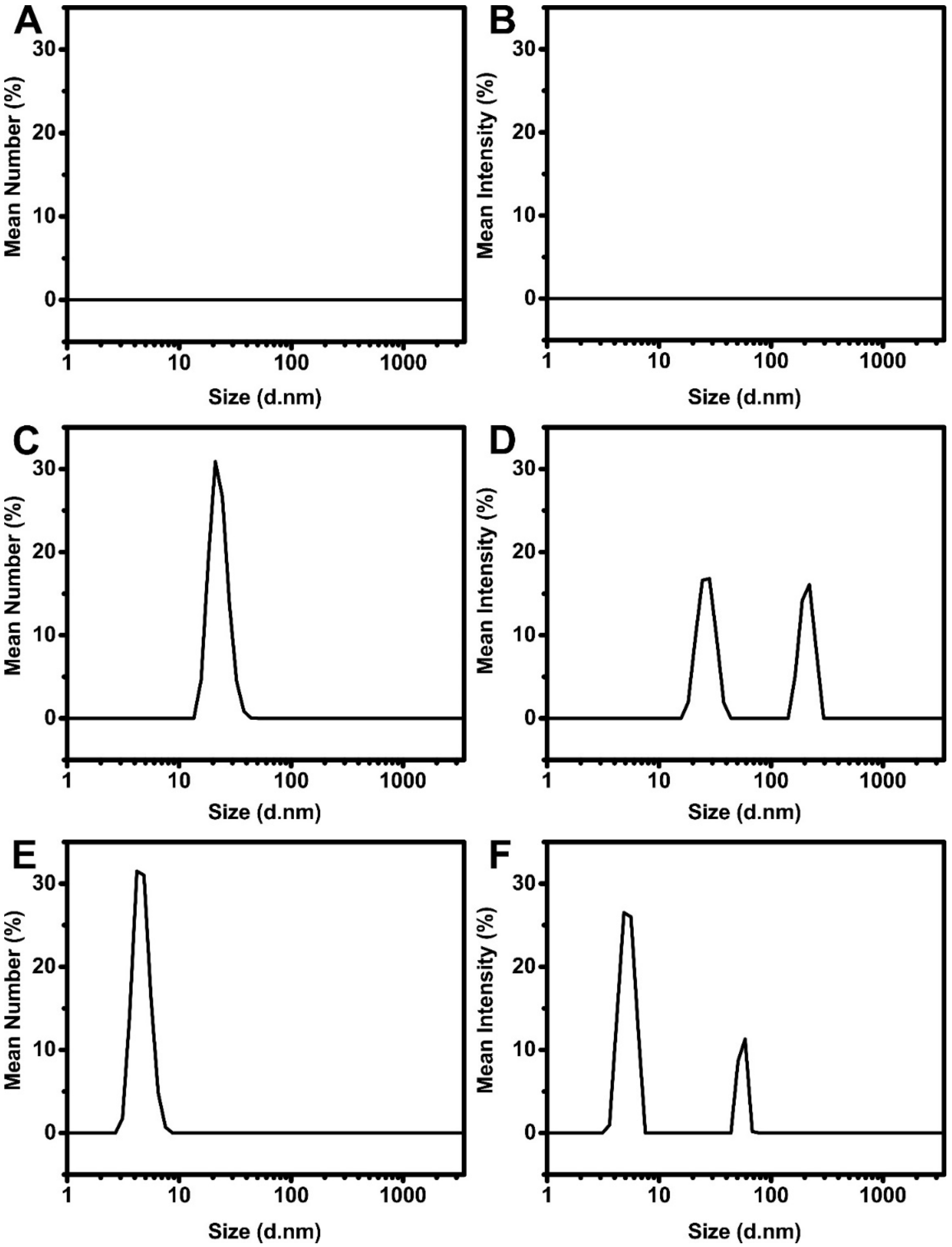

Figure S9: Size distributions from dynamic light scattering analysis of TPP, polyA, and **Met^{O}-*rac*-C$^{O}_{65}$** solutions. (A) Number and (B) intensity size distributions of a solution of 12 mM TPP in 150 mM NaCl at pH 7.0. (C) Number and (D) intensity size distributions of a solution of 5.0 mg/mL polyA in 150 mM NaCl at pH 7.0. (E) Number and (F) intensity size distributions of a solution of 3.0 mg/mL **Met^{O}-*rac*-C$^{O}_{65}$** in 150 mM NaCl at pH 7.0. d.nm = average hydrodynamic diameter in nanometers.

Synthetic Procedures

S-(*tert*-butylcarboxymethyl)-L-cysteine L-cysteine hydrochloride monohydrate (5.0 g, 28 mmol, 1.0 eq) and NaOH (2.5 g, 57 mmol, 2.0 eq) were dissolved in DI H_2O (40 mL) with stirring. The reaction mixture was cooled to 0 °C in an ice-water bath before the dropwise addition of *tert*-butyl bromoacetate (2.8 mL, 31 mmol, 1.1 eq) over 30 min. The ice bath was then removed and 10 mL of THF was added. The reaction mixture was stirred at room temperature overnight resulting in formation of a white solid precipitate. The while solid was collected via filtration and washed with a 3:7 mixture of 95% EtOH and diethyl ether. The solids were placed in a 50 mL centrifuge tube and washed 3 times with a 95:5 mixture of diethyl ether and methanol to remove residual impurities. The sticky white solid was then dried under vacuum to give the product. (4.1 g, 61%). Spectra data were in agreement with previously reported values.[1]

S-(*tert*-butylcarboxymethyl)-L-cysteine N-carboxyanhydride (tBuCM-Cys NCA)[1,2] S-(*tert*-butylcarboxymethyl)-L-cysteine (2.0 g, 8.5 mmol, 1.0 eq) was suspended in analytical grade THF (40 mL) in a heavy walled glass container. Epichlorohydrin (6.7 mL, 85 mmol 10 eq) was then added**.** In a well-ventilated fume hood, triphosgene (1.3 g, 4.6 mmol, 0.55 eq) was added, and the vessel was sealed and allowed to react for 2 h at room temperature. ***WARNING: Triphosgene is an extremely dangerous chemical and proper precautions must be taken to avoid exposure.*** The resulting turbid mixture was then cooled to 4 °C and 10 mL of cold DI H_2O was added. The reaction mixture was stirred for 1 minute before extraction with ethyl acetate, which was then washed with brine and dried with anhydrous sodium sulfate. The resulting solution was transferred to an oven dried Schlenk flask and the solvent was removed under vacuum. The crude product was transferred to a N_2 filled glove box, resuspended in minimal THF, and layered under 5x hexanes (v/v) to give white or slightly off-white crystals. (1.1 g, 50%). Spectral data were in agreement with previously reported values.[1]

Procedure for synthesis of poly(S-(*tert*-butylcarboxymethyl)-L-cysteine) ($\mathbf{C^{BCM}_{65}}$) Samples of $\mathbf{C^{BCM}_{65}}$ were prepared at *ca*. 500 mg scale in a N_2 filled glove box. bpyNiCOD initiator solution (3.1 mL, 20 mg/mL in THF) was quickly added to a solution of tBuCM-Cys NCA (10 mL, 50 mg/mL in THF). After *ca*. 90 min, complete consumption of NCA was confirmed by FTIR spectroscopy. In order to determine polypeptide chain lengths a small aliquot of the reaction mixture (*ca*. 200 µL) was removed for end-group analysis where active chain-ends were reacted with mPEG-NCO (*vide infra*). The remaining polypeptide solution was then end capped with excess acetic anhydride, precipitated by addition to DI H_2O (50 mL), and isolated by centrifugation. The solid was then washed two additional times with DI H_2O and dried under reduced pressure to yield $\mathbf{C^{BCM}_{65}}$ as a lightly purple colored powder. (460 mg, 92%) Spectral data were in agreement with previously reported values.[1]

General procedure for determination of polypeptide chain length using end-group analysis after reaction with mPEG-NCO[3] The procedure for synthesis of $\mathbf{C^{BCM}_{65}}$ was followed. Once the polymerization reaction was determined to be complete by FTIR, a solution of mPEG-NCO (M_n = 1000 Da, 50 mg/mL in THF, 4 eq per bpyNiCOD) was added in a N_2 filled glove box to a *ca*. 200 µL aliquot of active polymerization reaction mixture. The sample was let stand overnight, and then removed from the glove box and the polypeptide was precipitated by addition to DI H_2O. The sample was centrifuged at 3000 rpm and the supernatant was discarded. The pellet was washed 3 times with DI H_2O and centrifuged to remove unconjugated mPEG-NCO, and the resulting pellet

was then lyophilized to yield the PEG-polypeptide conjugate as a white solid (typical yields = 90 to 95%). To determine the molecular weight (M_n) of the polypeptide, a ^{1}H NMR spectrum was obtained in deuterated trifluoroacetic acid (TFA-d). The ratio of the integral of the methylene unit closest to the polypeptide backbone to the integral of the PEG methylene resonance was used to calculate polypeptide length (see spectral data section).

General procedure for synthesis of poly(S-carboxymethyl-L-cysteine), sodium salt (C^{CM}_{65})[1] A sample of $\mathbf{C^{BCM}_{65}}$ (450 mg) was dissolved in TFA (20 mg/mL) and allowed to stand for 5 h. The reaction mixture was diluted to 10 mg/mL by slowly adding 50 mM $NaHCO_3$ and then transferred to a 1000 Da MWCO dialysis bag and dialyzed against aqueous 50 mM $NaHCO_3$ (24 h, 3 dialyzate changes) followed by DI H_2O (24 h, 4 dialyzate changes). The retentate was then lyophilized to give the product as a white fluffy solid. (340 mg, 89%). Spectral data were in agreement with previously reported values.[1]

General procedure for synthesis of poly(dehydroalanine) (A^{DH}_{65}) A sample of $\mathbf{C^{CM}_{65}}$ (340 mg) was dissolved in 150 mM sodium phosphate buffer (pH 8) at a concentration of 20 mg/mL. Iodomethane (25 eq per S-(carboxymethyl)-L-cysteine residue) was added, and the reaction flask was sealed and placed in a heating block at 37 °C, covered in foil and allowed to react for 3 days. The resulting suspension was mixed with a solution of BHT in dinitrogen sparged DMSO (0.2 mg/mL) to give a final 0.01 mol% ratio of BHT to dehydroalanine residues and then transferred to a 2000 Da MWCO dialysis bag and dialyzed against dinitrogen sparged DI H_2O (48 h, 4 dialyzate changes, dialysis jar covered in foil). The retentate was then lyophilized to give the product as a white solid (130 mg, 100%). Spectral data were in agreement with previously reported values.[1]

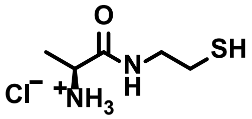

L-Alanine 2-mercaptoethylamide hydrochloride, 1a Prepared from N-Boc-L-alanine using general procedure for the synthesis of L-amino acid 2-mercaptoethylamides. Yield given in Table S1.
^{1}H NMR (400 MHz, D_2O, 25 °C) δ 4.11 (s, 1H), 3.7-3.4 (m, 2H), 3.0-2.6 (d, 2H), 1.6-1.5 (d, 3H)

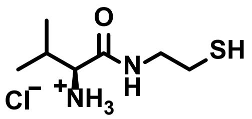

L-Valine 2-mercaptoethylamide hydrochloride, 1b Prepared from N-Boc-L-valine using general procedure for the synthesis of L-amino acid 2-mercaptoethylamides. Yield given in Table S1.
^{1}H NMR (400 MHz, D_2O, 25 °C) δ 3.8 (s, 1H), 3.78 (m, 1H), 3.55 (m, 1H), 3.0-2.7 (m, 1H), 2.2 (m, 1H), 1.05 (d, 6H)

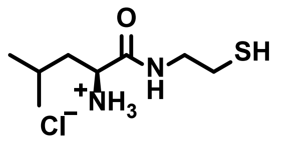

L-Leucine 2-mercaptoethylamide hydrochloride, 1c Prepared from N-Boc-L-leucine using general procedure for the synthesis of L-amino acid 2-mercaptoethylamides. Yield given in Table S1.

^{1}H NMR (400 MHz, D_2O, 25 °C) δ 4.05 (s, 1H), 3.78-3.35 (m, 2H), 3.0 -2.65 (m, 2H), 1.7 (m, 2H), 1.68 (m, 1H), 0.95 (s, 6H)

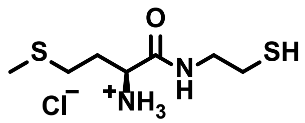

L-Methionine 2-mercaptoethylamide hydrochloride, 1d Prepared from N-Boc-L-methionine using general procedure for the synthesis of L-amino acid 2-mercaptoethylamides. Yield given in Table S1.

^{1}H NMR (400 MHz, D_2O, 25 °C) δ 4.2 (s, 1H), 3.8-3.3 (m, 2H), 2.9 (m, 1H), 2.75 (m, 1H), 2.7 (m, 1H), 2.22 (q, 2H) 2.18 (s, 3H)

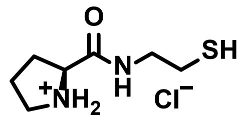

L-Proline 2-mercaptoethylamide hydrochloride, 1e Prepared from N-Boc-L-proline using general procedure for the synthesis of L-amino acid 2-mercaptoethylamides. Yield given in Table S1.

^{1}H NMR (400 MHz, D_2O, 25 °C) δ 4.25 (s, 1H), 3.55–3.45 (m, 2H), 3.3 (s, 2H) 2.8–2.5 (m, 2H), 2.35 (s, 1H), 1.90 (s, 1H), 1.85 (s, 2H)

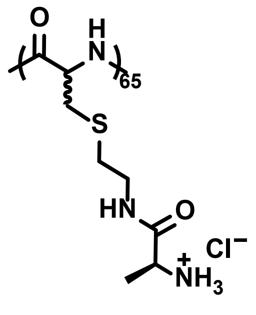

Ala-*rac*-C$_{65}$ Prepared from **1a** and **A$^{DH}_{65}$** using the General procedure for modification of **A$^{DH}_{65}$** with L-amino acid 2-mercaptoethylamides. Yield given in Table S1.

^{1}H NMR (400 MHz, D_2O, 25 °C): δ 4.7-4.3 (br s, 1H), 4.0 (br s, 1H), 3.45 (br s, 2H), 3.0 (br d, 2H), 2.7 (br s, 2H), 1.55-1.15 (br d, 3H)

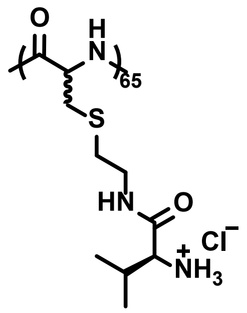

Val-*rac*-C_{65} Prepared from **1b** and **A^{DH}_{65}** using the General procedure for modification of **A^{DH}_{65}** with L-amino acid 2-mercaptoethylamides. Yield given in Table S1.

^{1}H NMR (400 MHz, D_2O, 25 °C): δ 4.45 (br s, 1H), 3.5 (br s, 1H), 3.4-3.2 (br d, 2H), 2.85 (br d, 2H), 2.55 (br s, 2H), 2.0 (br s, 1H), 0.85 (s, 6H)

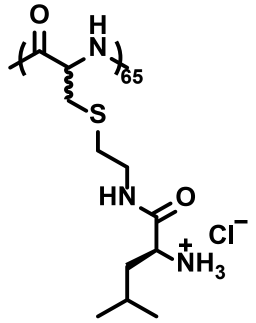

Leu-*rac*-C_{65} Prepared from **1c** and **A^{DH}_{65}** using the General procedure for modification of **A^{DH}_{65}** with L-amino acid 2-mercaptoethylamides. Yield given in Table S1.

^{1}H NMR (400 MHz, D_2O, 25 °C): δ 4.55 (br s, 1H), 3.95 (br s, 1H), 3.45 (br d, 2H), 2.95 (br d, 2H), 2.7 (br s, 2H), 1.65 (br s, 3H), 0.90 (s, 6H)

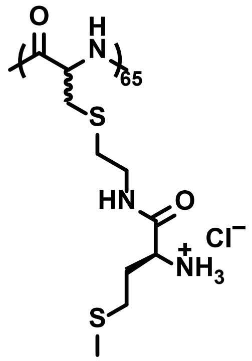

Met-*rac*-C_{65} Prepared from **1d** and **A^{DH}_{65}** using the General procedure for modification of **A^{DH}_{65}** with L-amino acid 2-mercaptoethylamides. Yield given in Table S1.

^{1}H NMR (400 MHz, D_2O, 25 °C): °C): δ 4.65 (br s, 1H), 3.90 (br s, 1H), 3.5 (br d, 2H), 3.1 (br d, 2H), 2.78 (br s, 2H), 2.62 (br s, 2H), 2.1 (br s, 5H)

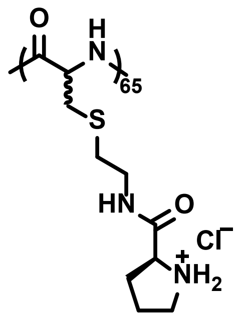

Pro-*rac*-C$_{65}$ Prepared from **1e** and **A^{DH}$_{65}$** using the General procedure for modification of **A^{DH}$_{65}$** with L-amino acid 2-mercaptoethylamides. Yield given in Table S1.
^{1}H NMR (400 MHz, D_2O, 25 °C): δ 4.5 (br s, 1H), 4.35 (s, 1H), 3.5 (br, 2H), 3.25-2.65 (br m, 4H), 2.65 – 2.4 (br d, 3H), 2.2 – 2.0 (br d, 2H), 1.75 (br s, 1H)

Analysis of chain length after modification of C^{BCM}$_{50}$ to A^{DH}$_{50}$ to Leu-*rac*-C$_{50}$ The procedure for synthesis of **C^{BCM}** was adapted to prepare **PEG$_{22}$-*b*-C^{BCM}$_{50}$**. After a small aliquot of the reaction mixture (*ca*. 200 µL) was removed for end-group analysis, a solution of mPEG$_{22}$-NCO (M_n = 1000 Da, 50 mg/mL in THF, 4 eq per bpyNiCOD) was added to the remaining polymerization reaction mixture in a N_2 filled glovebox. The general procedure for synthesis of poly(S-carboxymethyl-L-cysteine), sodium salt and the general procedure for synthesis of poly(dehydroalanine) were used to convert the product to **PEG$_{22}$-*b*-A^{DH}$_{50}$**. The procedure for modification of **A^{DH}** with L-amino acid 2-mercaptoethylamides was then followed using L-leucine 2-mercaptoethylamideto give **PEG$_{22}$-*b*-(Leu-*rac*-C)$_{50}$**. To compare average degrees of polymerization (DP) of the polypeptide segments, ^{1}H NMR spectra were obtained for **PEG$_{22}$-*b*-C^{BCM}$_{50}$** in d-TFA, **PEG$_{22}$-*b*-A^{DH}$_{50}$** in d$_6$-DMSO, and **PEG$_{22}$-*b*-(Leu-*rac*-C)$_{50}$** in D_2O. The ratio of the integral of the CH_2 group closest to the polypeptide backbone to the integral of the PEG CH_2 was used to calculate polypeptide length for **Peg$_{22}$-*b*-C^{BCM}$_{50}$**, the ratio of the integral of the alkene CH_2 to the integral of the PEG CH_2 was used to calculate the length for **PEG$_{22}$-*b*-A^{DH}$_{50}$**, and the ratio of the integral of the polypeptide backbone alpha-CH to the integral of the PEG CH_2 was used to calculate the length for **PEG$_{22}$-*b*-(Leu-*rac*-C)$_{50}$** (see Figure S1 and spectral data section). Negligible changes in DP were observed after each modification step.

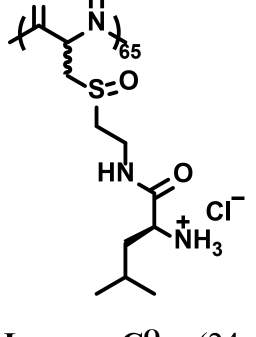

Leu-*rac*-C^{O}$_{65}$ (24 mg, 91.6%). ^{1}H NMR (400 MHz, D_2O, 25 °C): δ 4.6-4.2 (br s, 1H), 3.8 (br s, 1H), 3.7-2.7 (br m, 6H),1.55 (br s, 3H), 0.88 (s, 6H)

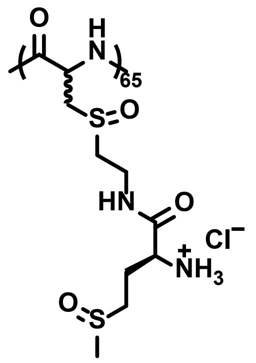

Met^{O}-*rac*-C^{O}$_{65}$ (25 mg, 91.5%). ^{1}H NMR (400 MHz, D_2O, 25 °C): δ 4.55 (br s, 1H), 3.78 (br s, 1H), 3.7-3.4 (br d, 2H), 3.1-2.95 (br d, 2H), 3.1 (br d, 2H), 2.9 (br s, 2H), 2.7 (br s, 3H), 2.1 (br s, 2H)

Spectral Data

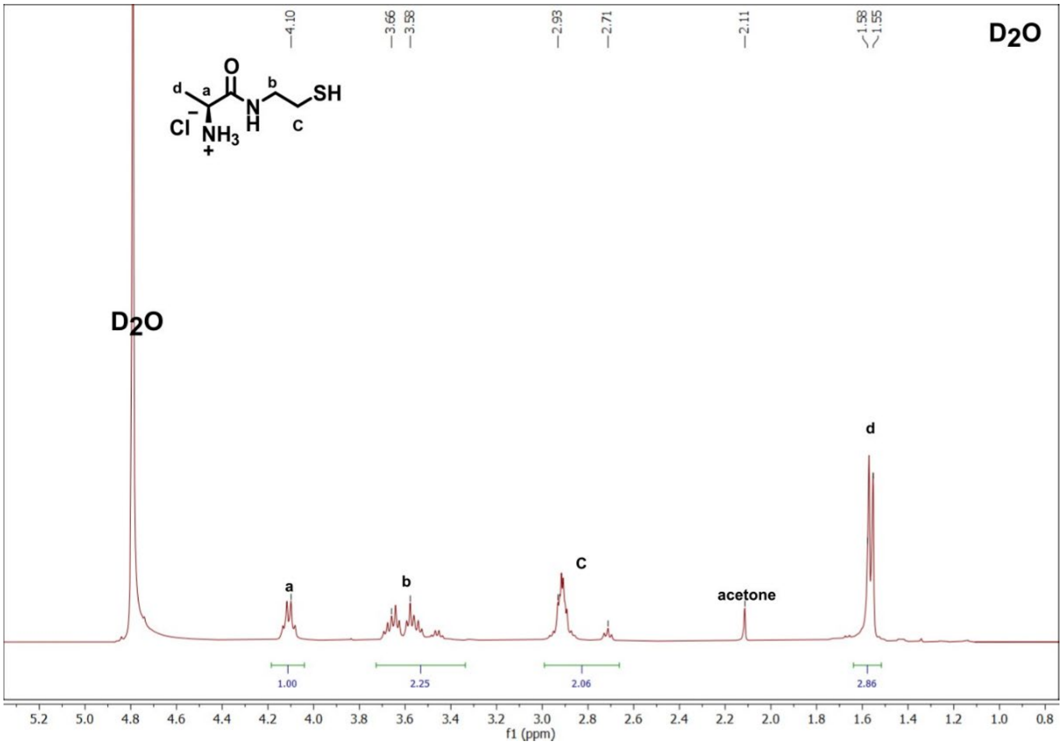

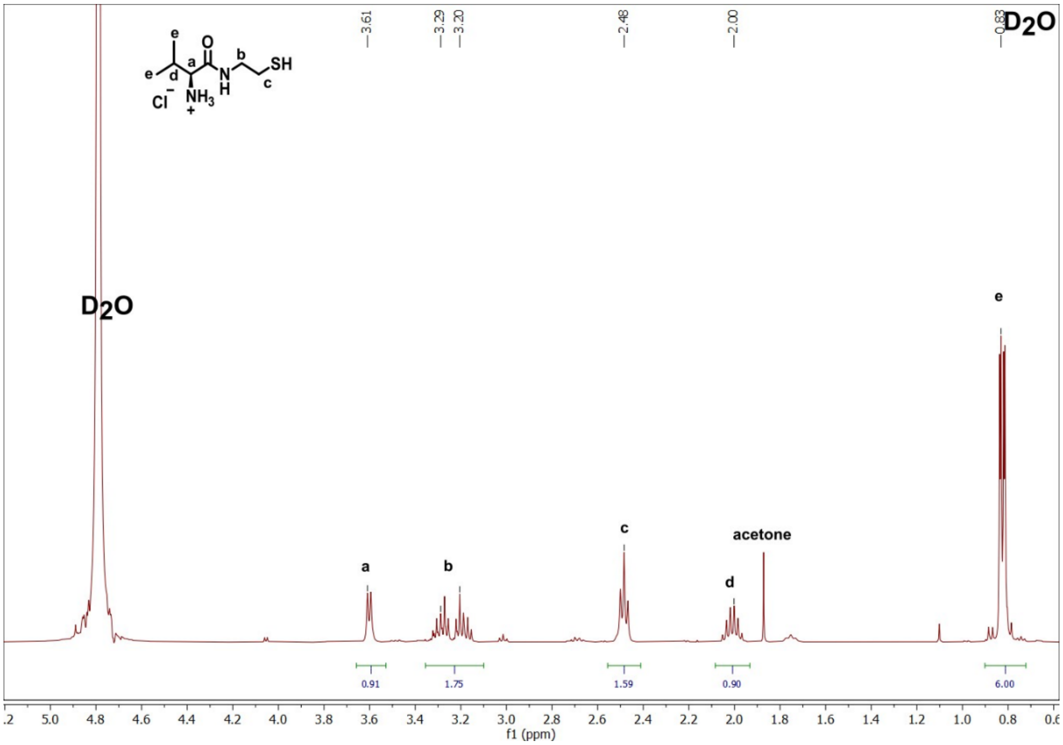

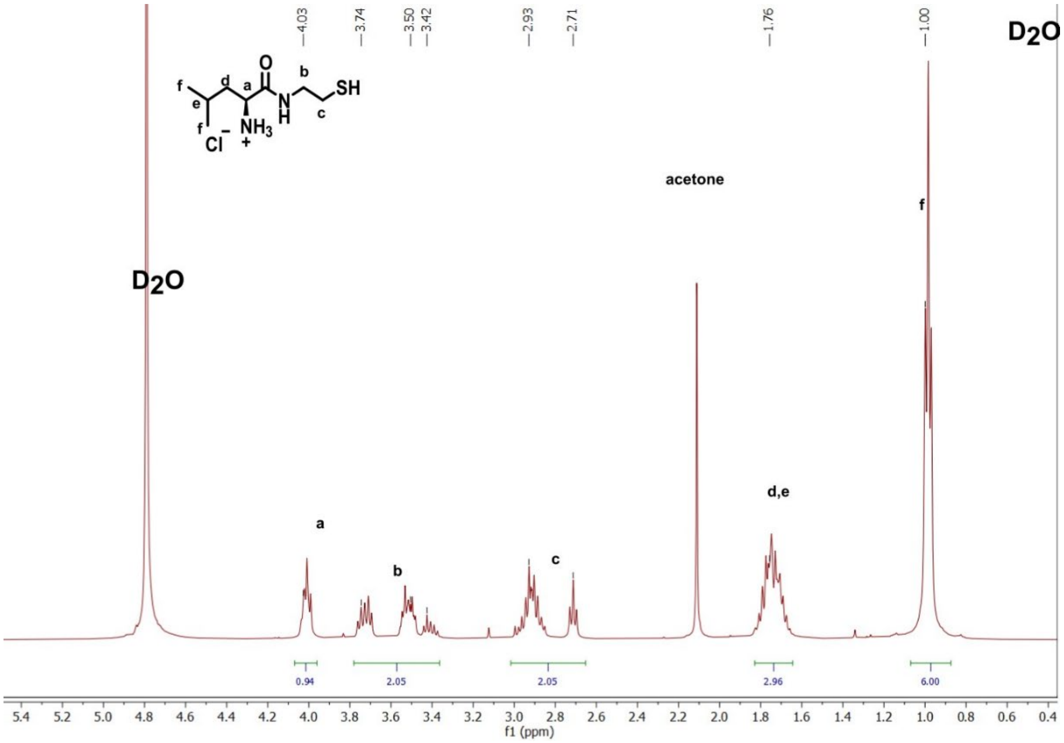

4.03
3.74
3.50
3.42
2.93
2.71
1.76
1.00
D2O
acetone
f
D2O
a
b
c
d,e
0.94
2.05
2.05
2.96
6.00
f1 (ppm)

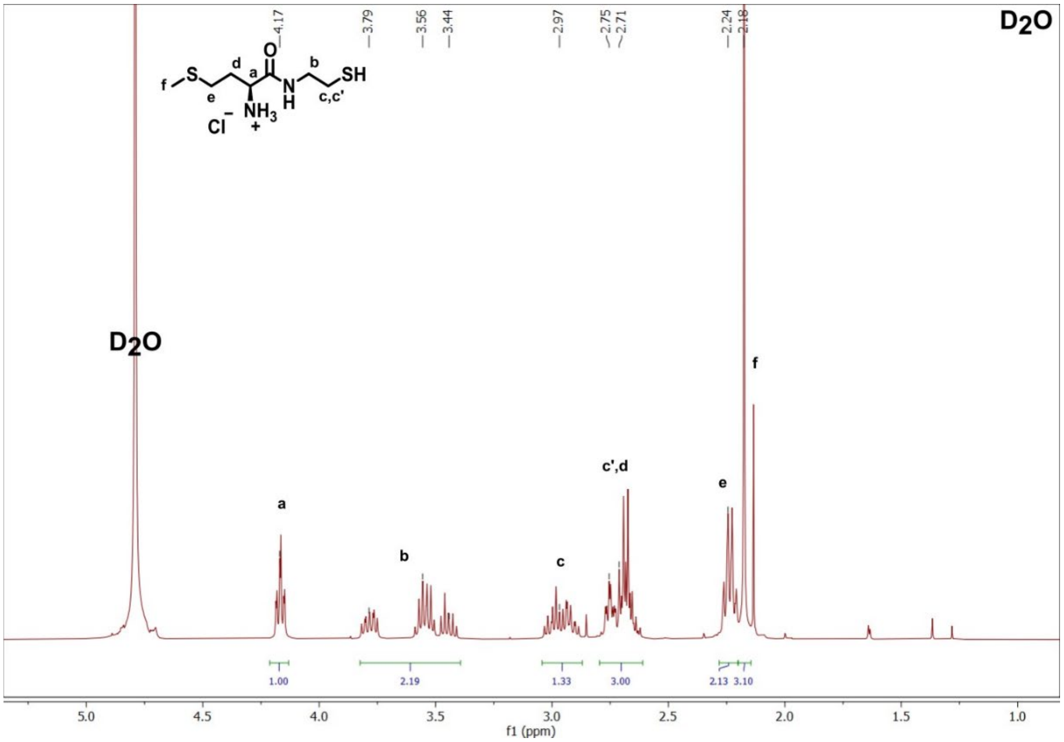

4.17
3.79
3.56
3.44
2.97
2.75
2.71
2.24
2.18
D2O
D2O
f
c',d
e
a
b
c
1.00
2.19
1.33
3.00
2.13
3.10
f1 (ppm)

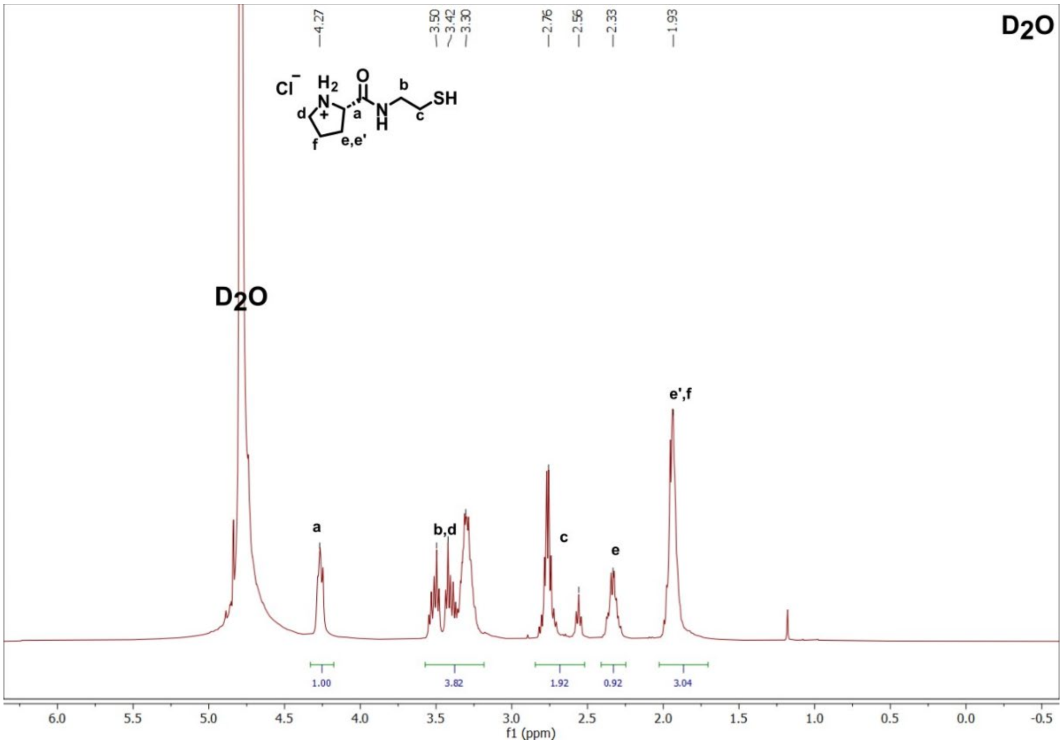
D2O
4.27
3.50
3.42
3.30
2.76
2.56
2.33
1.93
Cl⁻
H2
a
b
c
d
e,e'
f
SH
D2O
a
b,d
c
e
e',f
1.00
3.82
1.92
0.92
3.04
6.0
5.5
5.0
4.5
4.0
3.5
3.0
2.5
2.0
1.5
1.0
0.5
0.0
-0.5
f1 (ppm)

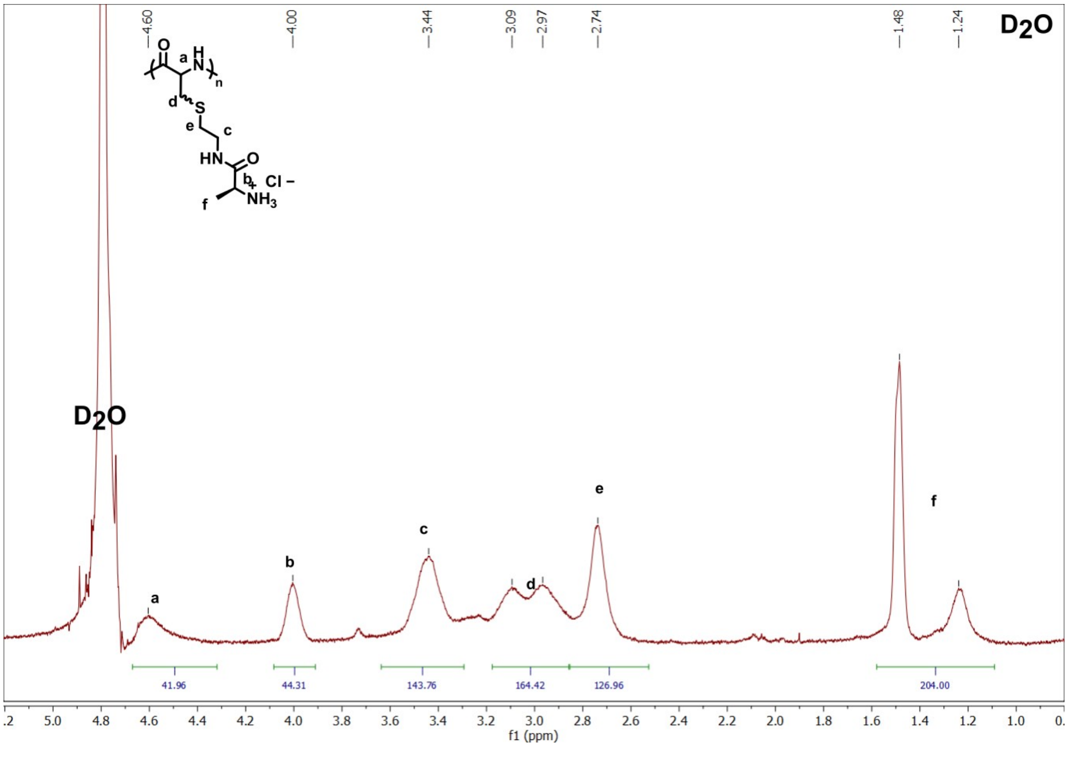
D2O
4.60
4.00
3.44
3.09
2.97
2.74
1.48
1.24
a
b
c
d
e
f
HN
Cl −
NH3
D2O
a
b
c
d
e
f
41.96
44.31
143.76
164.42
126.96
204.00
5.0
4.8
4.6
4.4
4.2
4.0
3.8
3.6
3.4
3.2
3.0
2.8
2.6
2.4
2.2
2.0
1.8
1.6
1.4
1.2
1.0
f1 (ppm)

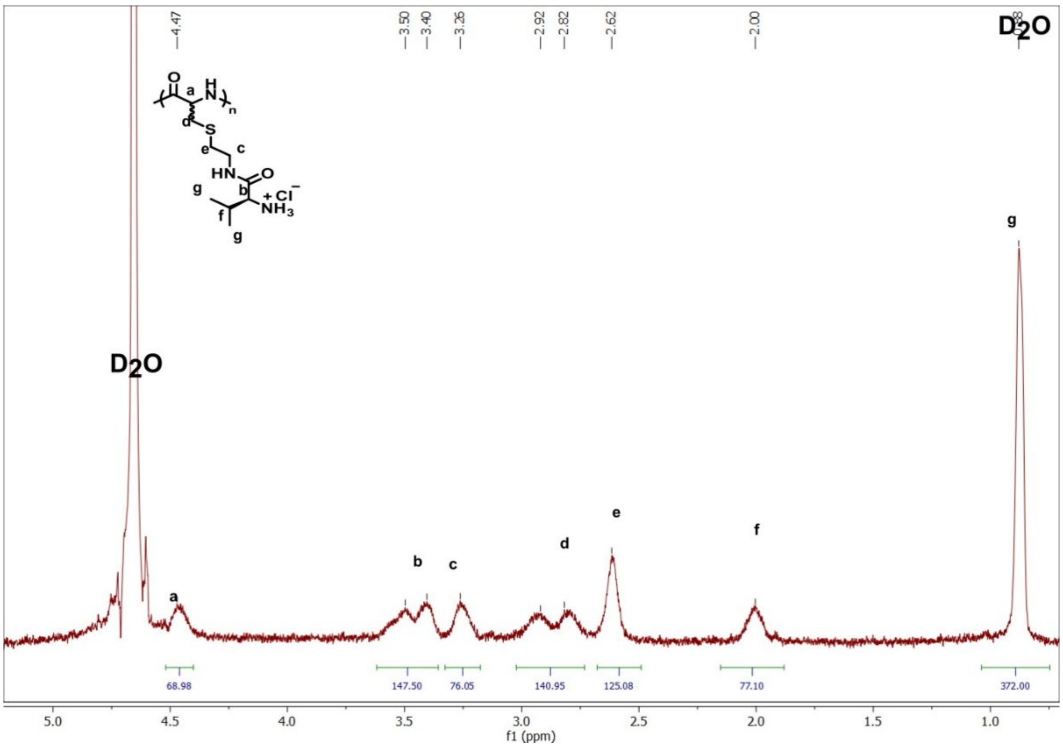
4.47
3.50
3.40
3.26
2.92
2.82
2.62
2.00
D2O
a
b
c
d
e
f
g
S
HN
O
+ Cl−
NH3
n
68.98
147.50
76.05
140.95
125.08
77.10
372.00
f1 (ppm)

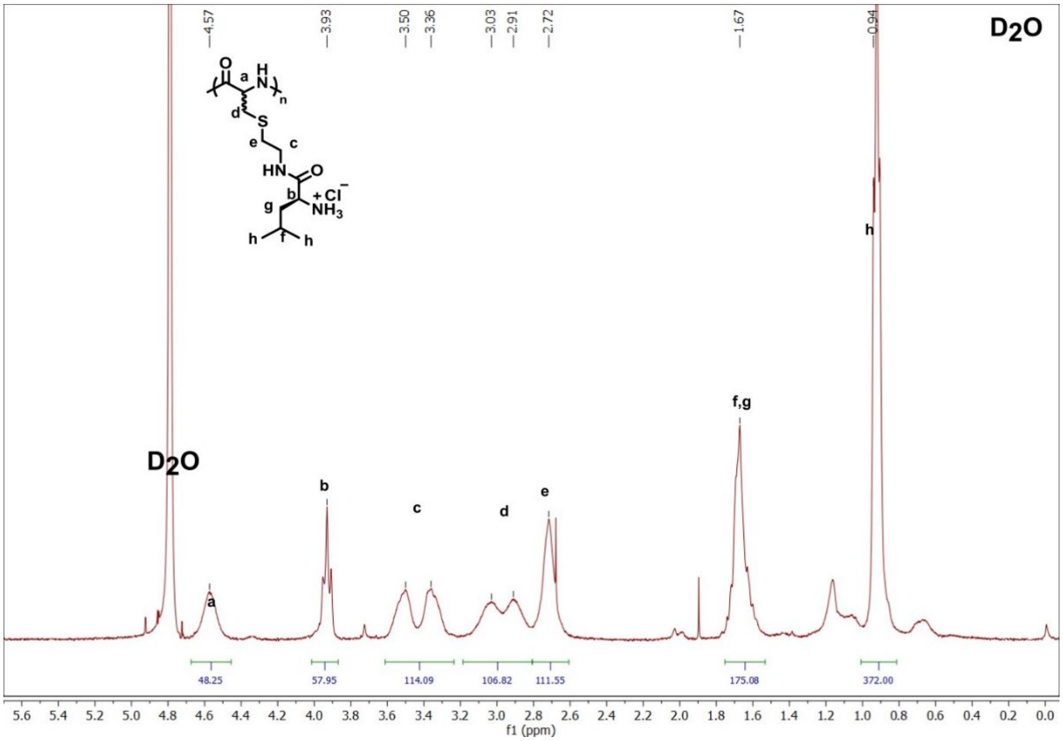
4.57
3.93
3.50
3.36
3.03
2.91
2.72
1.67
0.94
D2O
a
b
c
d
e
f,g
h
S
HN
O
+ Cl−
NH3
n
48.25
57.95
114.09
106.82
111.55
175.08
372.00
f1 (ppm)

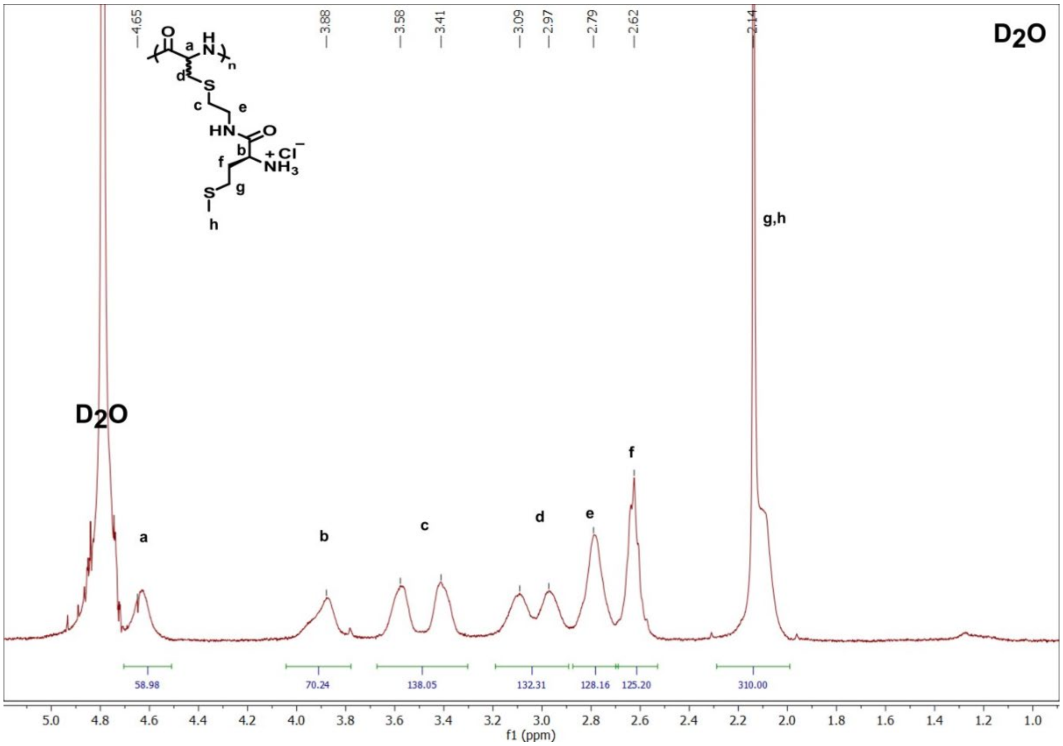
4.65
3.88
3.58
3.41
3.09
2.97
2.79
2.62
2.14
D2O
a
b
c
d
e
f
g
h
n
HN
NH3
Cl
D2O
g,h
58.98
70.24
138.05
132.31
128.16
125.20
310.00
f1 (ppm)

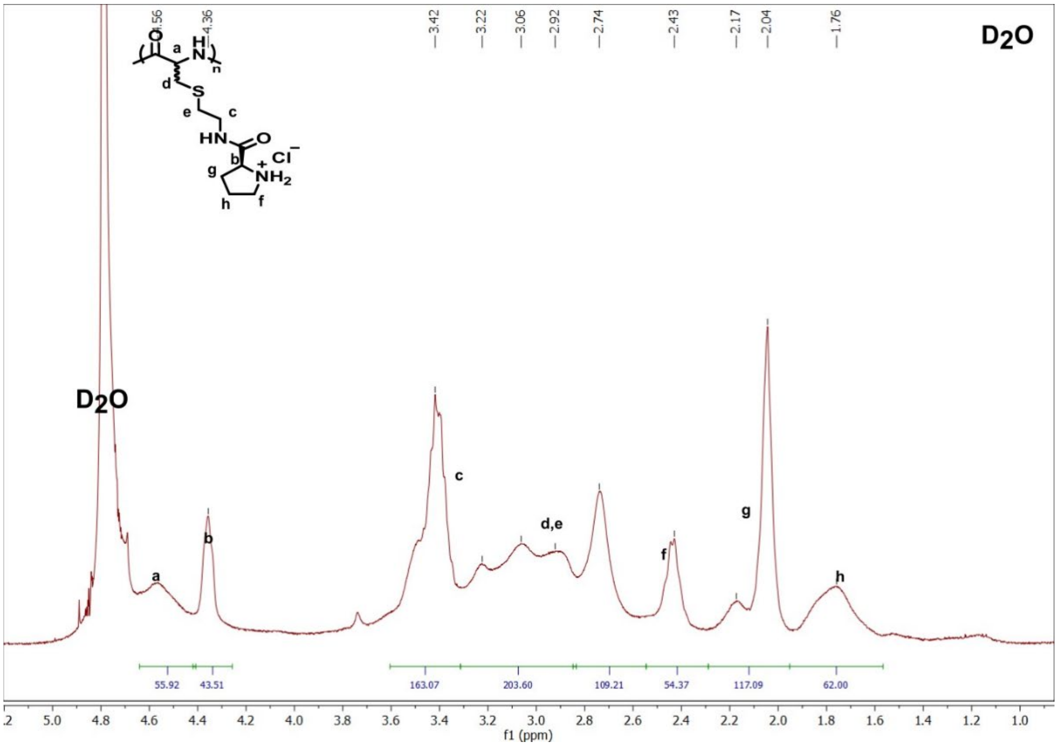
4.56
4.36
3.42
3.22
3.06
2.92
2.74
2.43
2.17
2.04
1.76
D2O
a
b
c
d
e
f
g
h
n
HN
NH2
Cl
D2O
d,e
55.92
43.51
163.07
203.60
109.21
54.37
117.09
62.00
f1 (ppm)

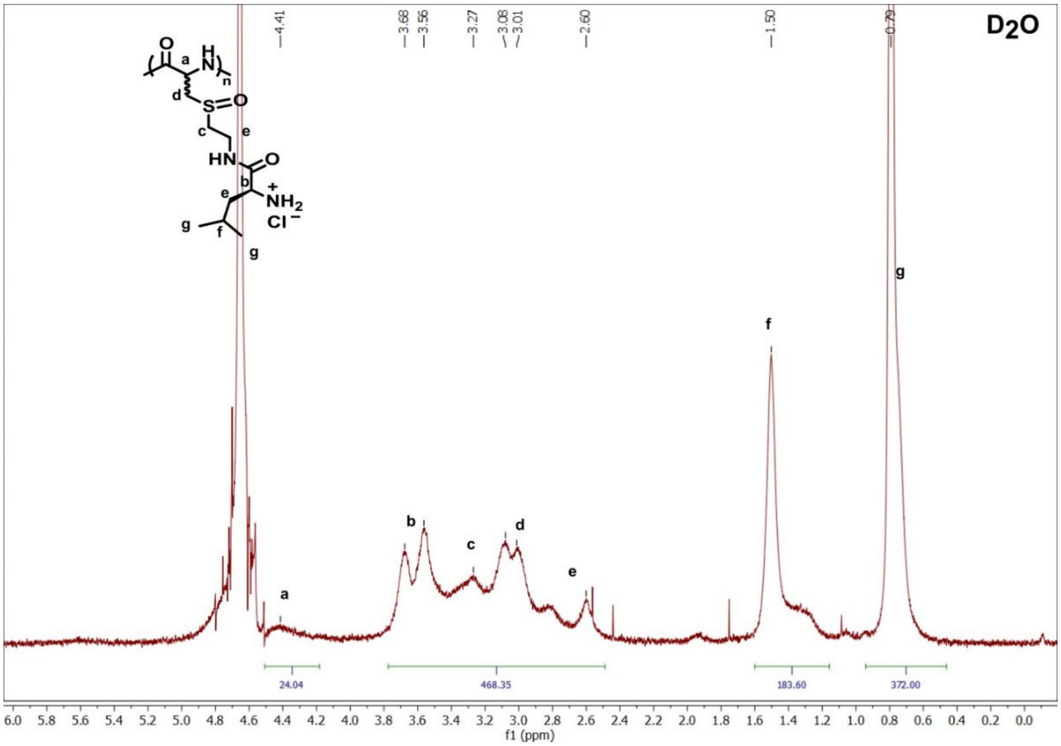
D2O
4.41
3.68
3.56
3.27
3.08
3.01
2.60
1.50
0.79
a
b
c
d
e
f
g
24.04
468.35
183.60
372.00
f1 (ppm)

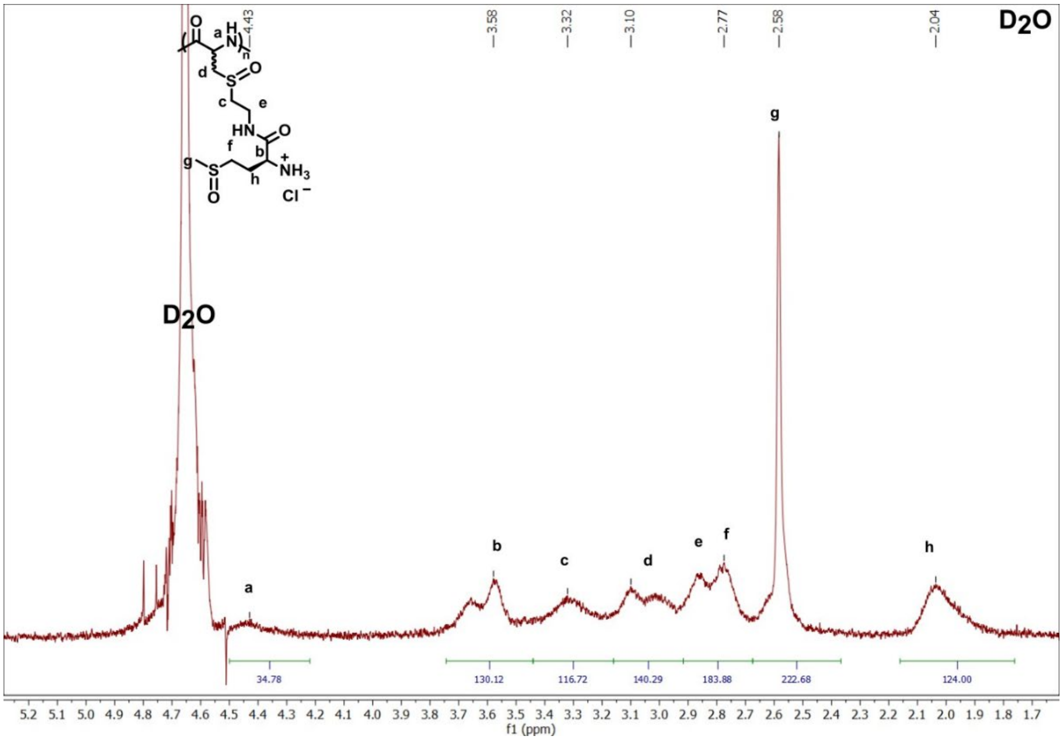
D2O
4.43
3.58
3.32
3.10
2.77
2.58
2.04
a
b
c
d
e
f
g
h
34.78
130.12
116.72
140.29
183.88
222.68
124.00
f1 (ppm)

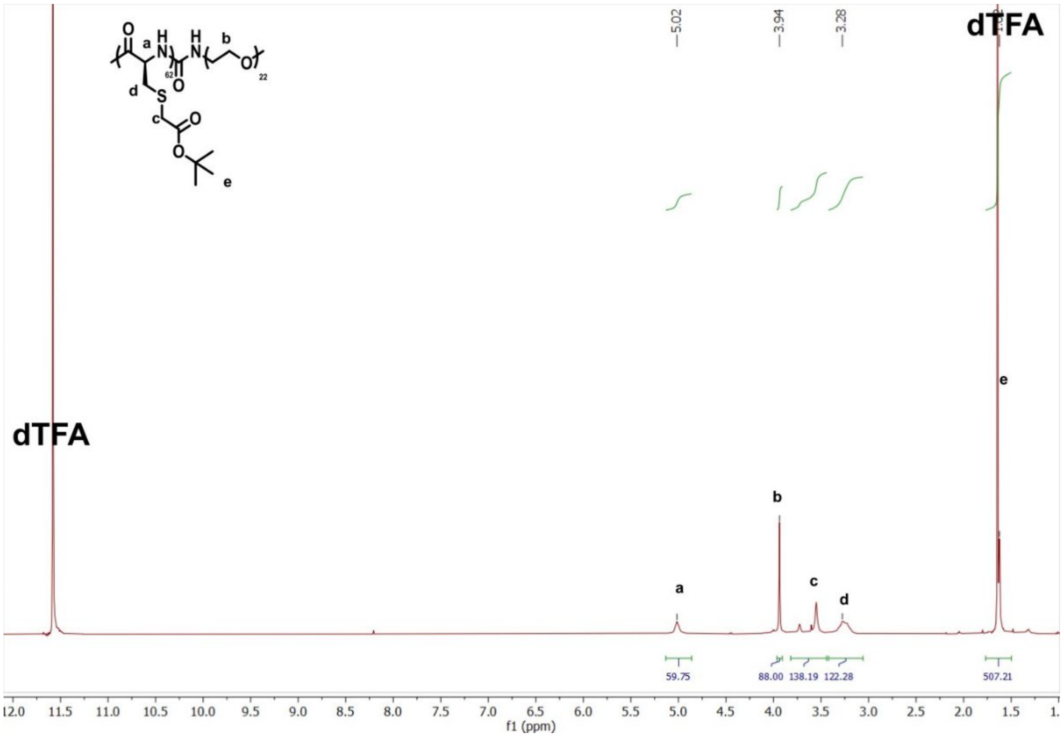
dTFA
dTFA
a
b
c
d
e
5.02
3.94
3.28
59.75
88.00
138.19
122.28
507.21
f1 (ppm)

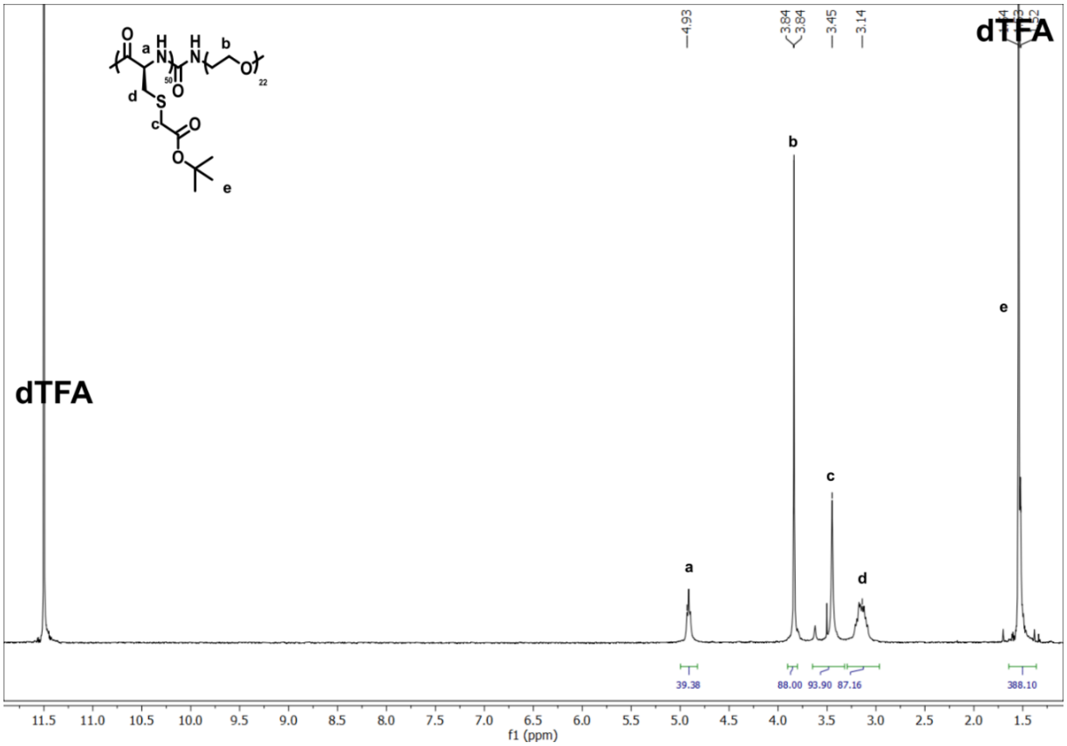
dTFA
dTFA
a
b
c
d
e
4.93
3.84
3.84
3.45
3.14
39.38
88.00
93.90
87.16
388.10
f1 (ppm)

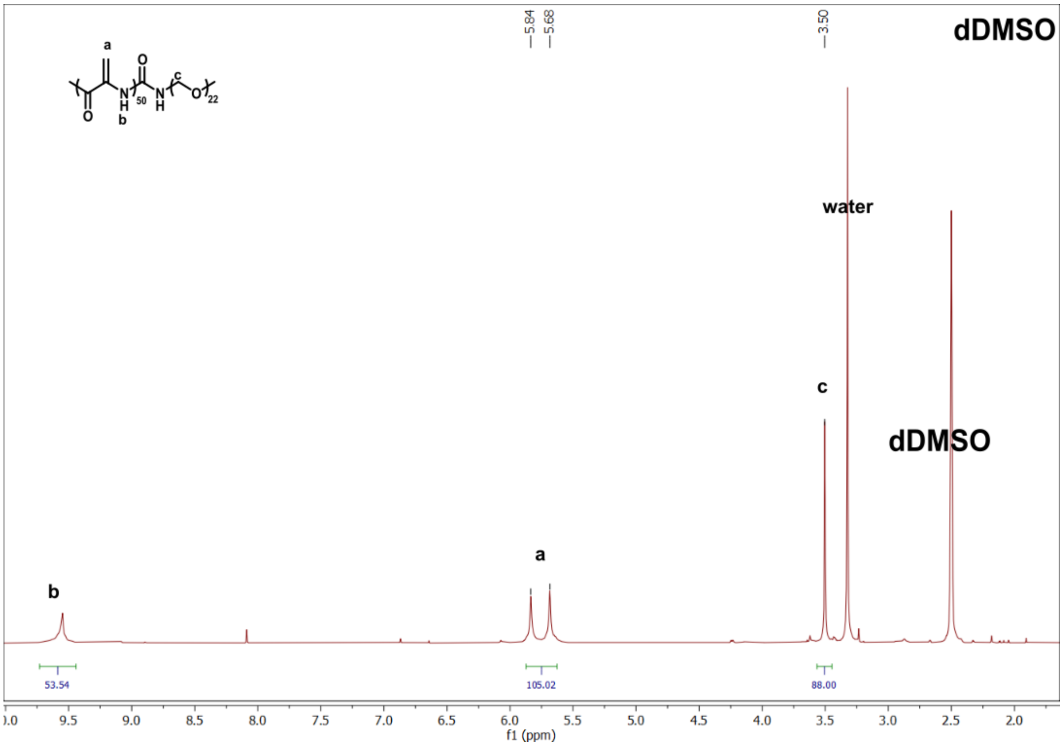
dDMSO
water
c
dDMSO
a
b
f1 (ppm)

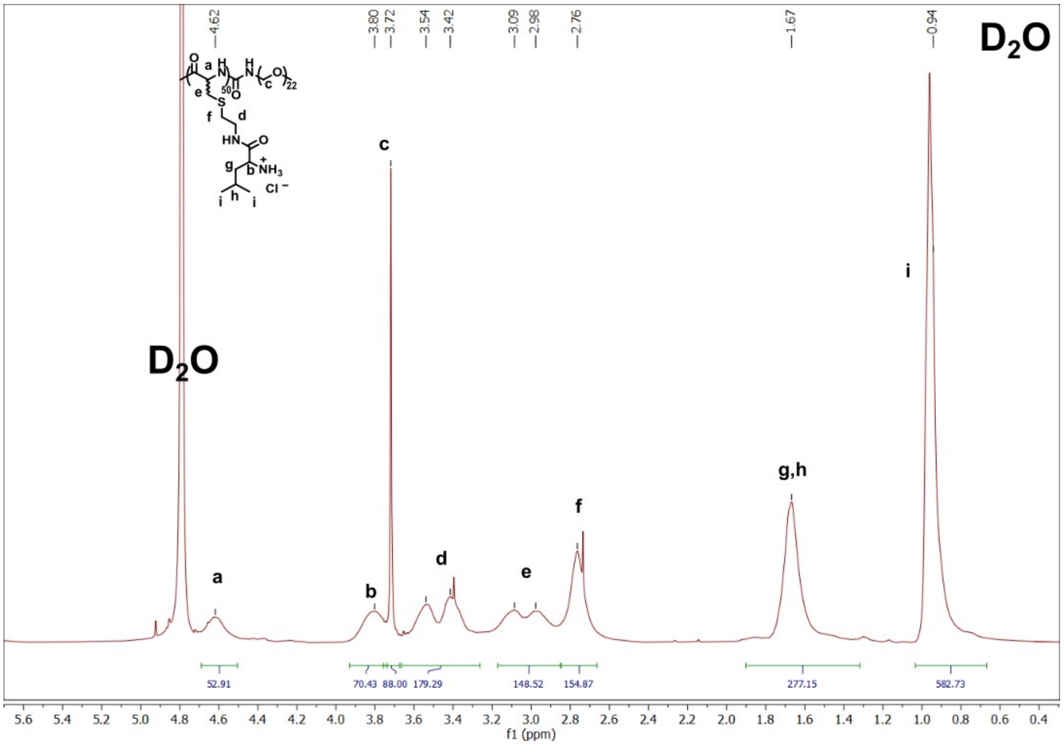
D2O
D2O
c
i
g,h
f
d
a
b
e
f1 (ppm)

References:

(1) Benavides, I.; Raftery, E. D.; Bell, A. G.; Evans, D.; Scott, W. A.; Houk, K. N.; Deming, T. J. Poly(Dehydroalanine): Synthesis, Properties, and Functional Diversification of a Fluorescent Polypeptide. *J. Am. Chem. Soc.* **2022**, *144* (9), 4214–4223. https://doi.org/10.1021/jacs.2c00383.

(2) Tian, Z.-Y.; Zhang, Z.; Wang, S.; Lu, H. A Moisture-Tolerant Route to Unprotected α/β-Amino Acid N-Carboxyanhydrides and Facile Synthesis of Hyperbranched Polypeptides. *Nat. Commun.* **2021**, *12* (1), 5810. https://doi.org/10.1038/s41467-021-25689-y.

(3) Brzezinska, K. R.; Curtin, S. A.; Deming, T. J. Polypeptide End-Capping Using Functionalized Isocyanates: Preparation of Pentablock Copolymers. *Macromolecules* **2002**, *35* (8), 2970–2976. https://doi.org/10.1021/ma011951f.
